# Supplementary material for: Rare genetic variants confer a high risk of ADHD and implicate neuronal biology
Source: Nature. 2025 Nov 12;649(8098):909–17. doi: 10.1038/s41586-025-09702-8 (PMC12823435; doi:10.1038/s41586-025-09702-8)
Supplement: Supplementary file 1 — Supplementary Notes, Supplementary Figs. 1–14 and Supplementary References. [file 41586_2025_9702_MOESM1_ESM.pdf]

nature portfolio

https://doi.org/10.1038/s41586-025-09702-8

Supplementary information

Rare genetic variants confer a high risk of ADHD and implicate neuronal biology

In the format provided by the authors and unedited

Supplementary Information

Rare coding variants confer high ADHD risk and implicate neuronal biology

Phenotype of individuals with class I variants in MAP1A, ANO8 or ANK2 ______________ 2

Validation in individuals with persistent ADHD ______________ 2

The impact of autism comorbidity ______________ 3

Immunoprecipitation-mass spectrometry (IP-MS) experiments ______________ 3

Common variant enrichment analysis of PPI-networks ______________ 8

Supplementary Figure 1. Overview of quality control of whole-exome sequencing data _ 10

Supplementary Figure 2. PCA plot of iPSYCH samples ______________ 11

Supplementary Figure 3. Mean load of variants across ADHD, controls and comorbid subgroups ______________ 12

Supplementary Figure 4. Sex-specific rare variant analyses ______________ 13

Supplementary Figure 5. Comorbidities of individuals with class I variants in MAP1A, ANO8 and ANK2 ______________ 14

Supplementary Figure 6. Ultra-rare variants in clinically ascertained individuals with persistent ADHD across pLI gene sets ______________ 14

Supplementary Figure 7. Sex-specific X chromosome rare variant analyses ______________ 15

Supplementary Figure 8. Expression of ADHD risk genes across brain developmental stages compared to neuronal expressed genes ______________ 16

Supplementary Figure 9. scDRS in cell types from prenatal human midbrain ______________ 17

Supplementary Figure 10. The association of rPTVs with education level and SES in individuals with ADHD and controls ______________ 18

Supplementary Figure 11. PCA plot of clinically ascertained individuals with persistent ADHD and controls ______________ 19

Supplementary Figure 12. Quantile-quantile plot of gene-based associations ______________ 19

Supplementary Figure 13. Ultra-rare class I variants in individuals with cardiovascular disease across iPSYCH ADHD risk gene sets ______________ 20

Supplementary Figure 14. iPSC-derived neurons recapitulate features of human neural progenitor cells (NPC) and excitatory neurons (ExN) in vitro. ______________ 21

Phenotype of individuals with class I variants in *MAP1A*, *ANO8* or *ANK2*

We identified 33 individuals carrying at least one rare class I variant in the three significant genes (*MAP1A*, *ANO8*, and *ANK2*) and conducted a detailed breakdown of their commodities. Specifically, we examined the presence of ADHD, ASD, schizophrenia (SZ), and intellectual disability (ID) diagnoses in these carriers. More than half of the carriers were comorbid with at least one of ASD, SZ, and/or ID (Supplementary Figure 5).

To explore disease severity further, we compared "age of ADHD diagnosis" of individuals who were carriers with non-carriers. No significant difference in age of ADHD diagnosis was identified (carrier ADHD: mean age at diagnosis = 12.6 years, standard error (s.e.) = 1.07; non-carrier ADHD: mean = 12.6 years, s.e. = 0.067; Wilcoxon rank-sum test, P value = 0.96**.**

Validation in individuals with persistent ADHD

We evaluated our findings in exome-sequencing data of German/Dutch samples including 1,078 clinically ascertained individuals with persistent ADHD and 1,738 controls. We focused on ultra-rare (i.e. singleton) variants to avoid potential biases introduced by origin of the data (see methods).

Overall, we identified a significantly increased load of ultra-rare class I variants (OR = 1.07, CI = [1.02, 1.11], P = 0.002) in individuals with ADHD compared to controls and the impact (the beta) was further increased when restricting to constrained genes (OR = 1.24, CI = [1.07, 1.45], P = 0.005), but no increased load of ultra-rare class II variants was observed (Supplementary Figure 6, Supplementary Table 5).

No single genes demonstrated significant increased burden of ultra-rare class I in ADHD compared to controls (Supplementary Table 7). In the clinical samples, ultra-rare class I variants were identified in seven out of the 20 genes ($P < 1x10^{-3}$) identified in analyses of iPSYCH and gnomAD samples, but none of them demonstrated significantly increased burden in individuals with ADHD. When testing sets of genes identified based on iPSYCH+gnomAD samples at different P-value thresholds (P< 0.001 (20 genes), 0.05 (316 genes), 0.1 (583 genes) (gene-sets listed in Supplementary Table 20), we identified increased burden of class I variants in ADHD cases compared to controls with a similar point estimate across gene-sets (Extended Figure 2; Supplementary Table 6), but only nominally significant for the gene sets identified at the two most lenient P-values (316 genes, OR = 1.475, CI=[1.01, 2.15], P = 0.042; 583 genes OR = 1.42, CI = [1.08, 1.86], P = 0.012). The point estimates exceed what was found for constrained genes which indicate that the genes identified based on iPSYCH+gnomAD samples represent

genes which tends to be more involved in ADHD than constrained genes. For comparison there was no increased load of ultra-rare synonymous variants in any of the gene sets (Supplementary Table 6).

It should be noted that exome-sequencing data from the majority of the controls (N= 1,663) used for the clinical samples are a part of gnomAD and thus, partly overlapping controls used for gene-discovery in iPSYCH samples. The control data was generated by the Myocardial Infarction Genetics Exome Sequencing Consortium: University of Lubeck, and since ADHD has been reported to be associated with increased risk of cardiovascular diseases, we performed a gene set burden test to test for load of class I variants in the three ADHD gene sets defined above comparing individuals with cardiovascular disease (N = 825) to those without (N = 838). No difference in the load was observed (Supplementary Figure 13, Supplementary Table 24).

The impact of autism comorbidity

We note that the prevalence of co-occurring ASD was higher in this study (37%) compared to the full population-based iPSYCH case-cohort sample (22%), which potentially could increase power to identify risk genes shared across ADHD and ASD. However, it has probably not influenced the gene discovery much since (i) the c-alpha results suggested substantial sharing of rare variant risk genes across ADHD and autism regardless of including comorbid ADHD-ASD cases, and (ii) the rare variant burden analysis across comorbidities showed no difference between ADHD with ASD and ADHD without comorbid conditions in the load of class I and class II variants in constrained genes (Extended Figure 4, Supplementary Table 19).

Immunoprecipitation-mass spectrometry (IP-MS) experiments

This section describes methods for generating the MAP1A and ANO8 IP-MS data in NPC and ExN.

Cell culture

Glutamatergic patterned induced excitatory neurons (ExN) were differentiated from a male (neurotypical doner) induced pluripotent stem cell (iPSC) line (iPS hDFn 83/22 iNgn2#9 [iPS3] in Nehme *et al.*[1]) by conditional doxycycline-inducible expression of the transcription factor NGN2 combined with small molecule-mediated developmental patterning as previously described[1]. A plate was coated with GelTrex (LifeTechnologies, A1413301) adhesion matrix (1:100 in DMEM/F:12, Life Technologies, Inc., 11320033) and cells were seeded at a density of 35,000 cells cm^{-2} in Stemflex media (Life Technologies, Inc., A3349401) containing Geneticin as selective antibiotic (Life Technologies, Inc., 10131027) (1:400). Once the cells

achieved 60% confluency, the monolayer was detached using Accutase (Life Technologies, Inc., A11105) and transferred onto new plates for cell expansion with rock inhibitor Y27632 (Stemgent, 04-0012). Cells were incubated at 37°C and 5% CO_2.

Differentiation of ExN

On Day 1, iPSC cells were differentiated in N2 media (Life Technologies, Inc.) supplemented with 10 µM SB431542 (Tocris, 1614), 2 µM XAV939 (Stemgent, 04-00046) and 100 nM LDN-193189 (Stemgent, 04-0074) along with doxycycline hyclate (2 µg mL^{-1}). Day 2 media was supplemented with N2+SB/XAV/LDN/doxycycline hyclate as previously described[1]. On Day 3, cell differentiation was continued in neurobasal media (Life Technologies, Inc.) supplemented with B27 (50X, Thermo Scientific), brain-derived neurotrophic factor (BDNF), ciliary neurotrophic factor (CTNF), glial cell-derived neurotrophic factor (GDNF) (R&D Systems 248-BD/CF, 257-NT/CF, and 212-GD/CF at 10 ng mL^{-1}) and doxycycline hyclate (2 µg mL^{-1}).

Marker gene expression plots

The snRNA-seq datasets of differentiating neurons spanning iPSC (day 0), NPC (day 4) and ExN (day 31) stages were previously described in [REF[2]]. The data were processed with CellRanger (v6.1.2)[3] using the refdata-gex-GRCh38-2020-A reference from 10x Genomics. CellRanger was run with the following parameters: --expect-cells=10000, --localmem=64, --nosecondary, --chemistry=SC3Pv3, and --include-introns. Nuclei with fewer than 300 detected genes and more than 5% mitochondrial genes were excluded from further analysis. For the remaining nuclei, the percentage of mitochondrial genes was included as a technical variable when performing data normalization using the SCTransform method in Seurat (v5.0.3)[4]. Data integration across multiple 10x runs were performed via STACAS (v2.2.2)[5], using 3000 anchor features, 30 dimensions, and cell type labels (iPSC, NPC, ExN) for semi-supervised alignment. UMAP projection plots showing log-transformed counts per million (CPM) expression for genes of interest were generated using Seurat.

Immunofluorescence (IF)

Cells were plated on 96-well microplates (PhenoPlates, Revvity) at the iPSC stage. On the day of the experiment, cells were washed with PBS and then fixed with 2% formaldehyde for 15 min, followed by 5 min of permeabilisation in 0.4% Triton X-100 (TBST). Cells were briefly washed with PBS before blocking with a 1% BSA-TBST solution for 30 min. Primary antibody dilutions were prepared in blocking solution. Cells were incubated with primary antibodies

overnight in a humid chamber at 4°C. Slides were washed three times in PBS. Secondary antibodies and conjugated antibodies were diluted in blocking solution and incubated with cells for 45 min in a humid chamber at room temperature. After incubation, wells were washed twice with PBS before incubating with 4,6-diamidino-2-phenylindole (DAPI) for 10 minutes. Before imaging, cells were washed three times with PBS. All antibodies used are listed below.

**Gene ID**	**Vendor & Catalog**	**Host & Clonality**	**Usage**	**Amount in IF**
**NeuN**	Abcam, ab104224	Mouse Monoclonal	Primary	1:1000
**MAP2**	Abcam, ab183830	Rabbit polyclonal	Primary	1:1000
**CUX2**	Proteintech, 82933-1-RR	Rabbit polyclonal	Primary	1:1000
**TUJ1**	Invitrogen, MA1-118	Mouse monoclonal	Primary	1:1000
**TUJ1**	Invitrogen, 53-4510-82	Mouse Monoclonal	Conjugated Antibody	1:500
**Homer1**	Synaptic Systems, 160003	Rabbit polyclonal	Primary	1:1000
**Synaptophysin**	Synaptic systems, 101308	Guinea Pig	Primary	1:1000
**Vimentin**	Abcam, ab24525	Rabbit Polyclonal	Primary	1:1000
**MSl1**	Proteintech, 13512-1-AP	Rabbit Polyclonal	Primary	1:1000
**MAP2**	Abcam, ab302547	Rabbit Monoclonal	Conjugated Antibody	1:500
**MAP2**	Abcam, ab225316	Rabbit Monoclonal	Conjugated Antibody	1:500
**Neun**	NBP1-77686AF488	Rabbit Polyclonal	Conjugated Antibody	1:500
**Goat Anti-Rabbit IgG H&L (Alexa Fluor® 488)**	Invitrogen, # A-11008	Goat Polyclonal	Secondary Antibody	1:3000
**Goat Anti-Mouse IgG H&L (Alexa Fluor® 488)**	Invitrogen, #A28175	Goat Recombinant Superclonal	Secondary Antibody	1:3000
**Goat Anti-Rabbit IgG H&L (Alexa Fluor® 594)**	Invitrogen, # A-11012	Goat Polyclonal	Secondary Antibody	1:3000
**Goat Anti-Mouse IgG H&L (Alexa Fluor® 594)**	Invitrogen, #A-11032	Goat Polyclonal	Secondary Antibody	1:3000
**Goat anti-Chicken IgY (H+L) Secondary Antibody, Alexa Fluor® 647**	Invitrogen, #A-21449	Goat Polyclonal	Secondary Antibody	1:3000

**Goat anti-Guinea Pig IgG (H+L) Highly Cross-Adsorbed Secondary Antibody, Alexa Fluor® 488**	Invitrogen #A-11073	Goat Polyclonal	Secondary Antibody	1:3000
**Goat anti-Guinea Pig IgG (H+L) Highly Cross-Adsorbed Secondary Antibody, Alexa Fluor® 647**	Invitrogen #A-21450	Goat Polyclonal	Secondary Antibody	1:3000

Fluorescence images acquisition

Cells were imaged on an Opera Phenix High-Content Screening System (PerkinElmer) using Harmony software (v4.9). Initial setup was in wide-field mode with a water-immersion 20× objective. For data acquisition, at least 20 fields per well were captured in confocal mode, acquiring a minimum of six Z-stacks per field at 2 μm intervals. Optimal exposure times for each field and channel were determined manually and then applied uniformly across all experiments. Image processing and refinement were performed in Fiji/ImageJ.

Protein extraction

Total protein extract was obtained by harvesting cells and either processing them immediately or snap-freezing them on dry ice for storage at -80°C. In both cases, cell pellets were washed with PBS and resuspended in 10x packed cell volume (PCV) IP lysis buffer (Thermo Scientific), with freshly added Halt protease and phosphatase inhibitors (Thermo Scientific). After a 20 min incubation time at 4°C, cells were collected by centrifugation (16,200 g, 20 min, 4°C) and resuspended in 3x PCV lysis buffer. The concentration of the samples was quantified using the Thermo BCA protein assay and when not used immediately, samples were stored at -80°C.

Immunoprecipitations (IP)

For each individual experiment, 2-3 mg of protein extract from freshly lysed cells differentiated in the same batch was incubated at 4°C overnight in the presence of 1-2 μg of the relevant antibody (see table below). For NPC IPs, cells were harvested at Day 4 of differentiation, for ExN IPs, cells were harvested at Day 31 of differentiation. On the next day, 50 μL of Protein A/G beads (Pierce) were added to each sample and incubated at 4°C for 4 hours. Flow-through was collected and beads were washed once with 1 mL lysis buffer (Pierce) supplemented with Halt protease and phosphatase inhibitors (Thermo Scientific), and twice with PBS. Beads were resuspended in 100 μL of PBS and 10% of the volume was employed for immunoblotting (see below), after being boiled in 6xSMASH buffer (50 mM Tris HCl pH 6.8, 10% Glycerol, 2%

SDS, 0.02% bromophenol blue, 1% b-mercaptoethanol) for 10 min at 95ºC. The remaining volume of the immunoprecipitate was stored at -80°C and subsequently used for mass spectrometry analysis.

Gene ID	Vendor & Catalog	Host & Clonality	Amount in IP	Amount in WB
**MAP1A**	Fortis: A301-444A	Rabbit polyclonal	2 ug	1:1000
**ANO8**	Biorbyt: orb394715	Rabbit polyclonal	2 ug	1:1000

Immunoblotting

Samples for immunoblotting were stored in 6x SMASH buffer (50 mM Tris HCl pH 6.8, 10% glycerol, 2% SDS, 0.02% bromophenol blue, 1% b-mercaptoethanol), boiled for 10 min at 95°C, separated on a NuPAGE 4-12% Bis-Tris Protein Gel (Invitrogen), and transferred onto a PVDF membrane (Life Technologies) by wet transfer (100 V for 2 hours). Membranes were blocked by incubation for one hour at room temperature in 10 mL TBS and 0.1% Tween (TBST) with 5% w/v BioRad Blotting-grade Blocker. Blots were incubated overnight at 4°C with the same primary antibody used for the immunoprecipitation, washed 3 times for 10 min with TBST and incubated for 45 min with a isotype-matching secondary antibody conjugated to horseradish peroxidase (Rabbit IgG polyclonal, Abcam ab37415). After washing 3 times for 5 min with TBST, bands were visualized using SuperSignal^{TM} West Femto Maximum Sensitivity Substrate (Thermo Scientific).

Mass spectrometry (MS)

IP samples on beads were washed at least five times with 100 µl 50 mM ammonium bicarbonate then 5 µl (200ng/ul) of modified sequencing-grade trypsin (Promega, Madison, WI) was spiked in and the samples were placed in a 37ºC room overnight. The samples were then centrifuged or placed on a magnetic plate if magnetic beads were used and the liquid removed. The extracts were then dried in a speed-vac (~1 hr). Samples were then re-suspended in 50 µl of HPLC solvent A (2.5% acetonitrile, 0.1% formic acid) and desalted by STAGE tip[6].

On the day of analysis the samples were reconstituted in 10 µl of HPLC solvent A. A nano-scale reverse-phase HPLC capillary column was created by packing 2.6 µm C18 spherical silica beads into a fused silica capillary (100 µm inner diameter x ~30 cm length) with a flame-drawn tip[7]. After equilibrating the column 4 µl of each sample was loaded via a Famos auto sampler

(LC Packings, San Francisco CA) onto the column. A gradient was formed and peptides were eluted with increasing concentrations of solvent B (97.5% acetonitrile, 0.1% formic acid). As peptides eluted they were subjected to electrospray ionization and then entered into an LTQ Orbitrap Velos Pro, Exploris 480, or Fusion Lumos mass spectrometer (Thermo Fisher Scientific, Waltham, MA). Peptides were detected, isolated, and fragmented to produce a tandem mass spectrum of specific fragment ions for each peptide.

Peptide sequences (and hence protein identity) were determined by matching the UniProt human protein database (release 2023_01) with the acquired fragmentation pattern using Sequest (Thermo Fisher Scientific, Waltham, MA)[8]. The database included a reversed version of all the sequences and the data were filtered to between 1-2% peptide false discovery rate. Protein quantification was performed using GFY Core Version 3.8 (Harvard University, Cambridge, MA).

IP-MS data analysis

Starting with protein quantification data for each IP-MS dataset (consisting of bait vs. IgG control IPs in triplicate), we performed the following steps: (1) $\log_2$ transform and median normalize protein intensities in each sample; (2) remove contaminants and proteins with no human gene name, detected with < 2 unique peptides, or detected in < 2 bait IP samples; (3) impute missing values for the remaining proteins in each sample by drawing from a normal distribution with mean of μ - 1.8σ and standard deviation of 0.3σ, where μ and σ are the mean and standard deviation of the observed protein intensities, respectively[9]; (4) calculate replicate correlations among bait and control IPs; (5) perform limma-based[10] (v3.54.2) two-tailed two-sample moderated t-test as implemented in the Genoppi R package (development branch, v1.1.0)[11], to calculate enrichment statistics ($\log_2$ fold change [FC], p-value, and false discovery rate [FDR]) of each protein in bait vs. control IPs; (6) define proteins with $\log_2$ FC > 0 and FDR ≤ 0.1 as significant interactors of the bait protein; (7) identify overlap between the significant interactors vs. known interactors in the InWeb PPI database[12].

Common variant enrichment analysis of PPI-networks

The variant-to-gene annotation file generated from human fetal brain Hi-C data[13] were downloaded from the H-MAGMA[14] GitHub webpage (https://github.com/thewonlab/H-MAGMA); we mapped the Ensembl gene IDs in the file to gene names using BioMart (Ensembl 109)[15] and only kept protein-coding genes expressed in the neuronal background (see PPI-network analyses in Methods) for downstream analysis. GWAS summary statistics for ADHD[16], autism spectrum disorder[17], bipolar disorder[18], major depressive disorder[19], and

schizophrenia[20] were obtained from the Psychiatric Genomics Consortium (https://www.med.unc.edu/pgc/download-results/). The summary statistics file for height[21] was obtained from the GIANT Consortium (https://portals.broadinstitute.org/collaboration/giant/index.php/GIANT_consortium_data_files). For each GWAS phenotype, we computed gene-based p-values using the SNP-wise mean model in MAGMA[22] (v1.09), using the 1000 Genomes[23] (phase3) EUR panel, and the H-MAGMA annotation file described above. Then we performed MAGMA gene-set analysis for each PPI network, computing a one-tailed P-value to assess whether the network genes are significantly associated with the GWAS phenotype compared to other protein-coding genes expressed in neurons.

Supplementary Figure 1. Overview of quality control of whole-exome sequencing data

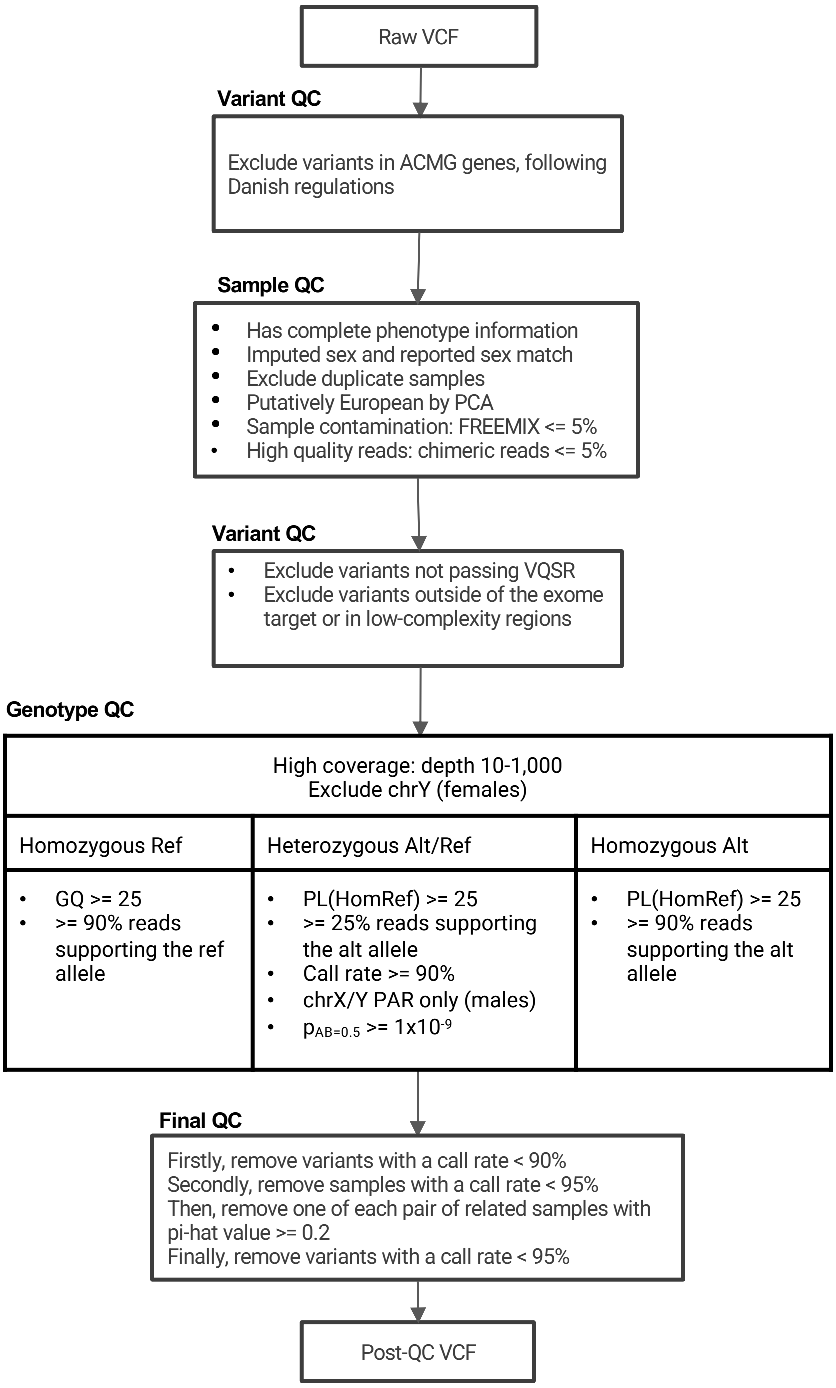

Overview of quality control steps at individual and variant level, implemented to ensure high quality data in the final VCF. Flow diagram showing sequential filtering steps applied to raw variant call format (VCF) files. Variants in ACMG-listed genes were removed in accordance with Danish regulations. Samples were retained only if phenotype data were complete, imputed sex matched reported sex, duplicates were excluded, ancestry was classified as putatively European by principal component analysis (PCA), contamination was ≤5% (FREEMIX), and chimeric reads were ≤5%. Variants not passing variant quality score recalibration (VQSR) and those outside the exome target or in low-complexity regions were excluded. Additional genotype-, variant- and sample-level filters were applied. GQ, genotype quality. PAR, pseudo autosomal region.

Supplementary Figure 2. PCA plot of iPSYCH samples

a.

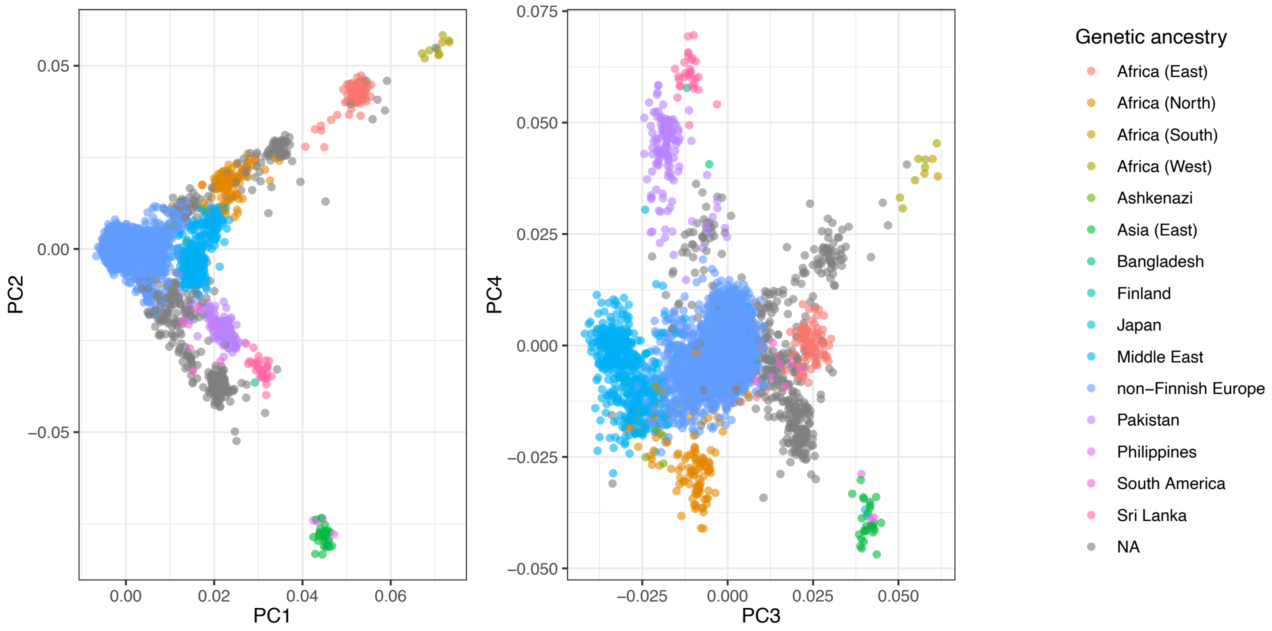

b.

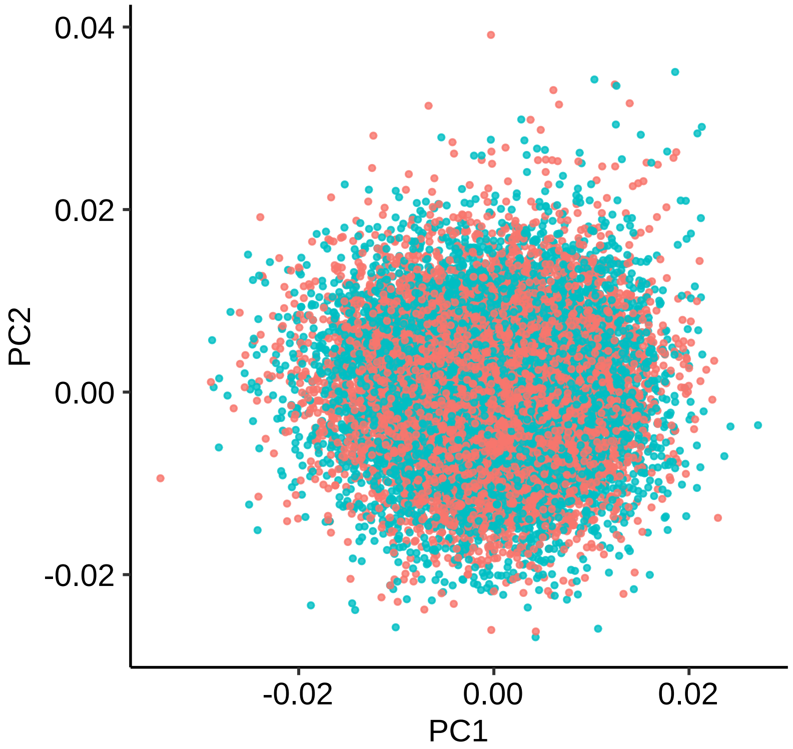

PCA plot of iPSYCH samples **a.** before quality control (11,051 with ADHD and 10,448 controls), individuals are marked in colour depending on the ancestry group they have been assigned to in the PCA (See Supplementary Table 23, for sample sizes of non-European ancestries). **b.** After quality controls individuals with ADHD (N = 8,895) marked in red and controls (N = 9,001) marked in light blue. PC, principal component.

Supplementary Figure 3. Mean load of variants across ADHD, controls and comorbid subgroups

a.

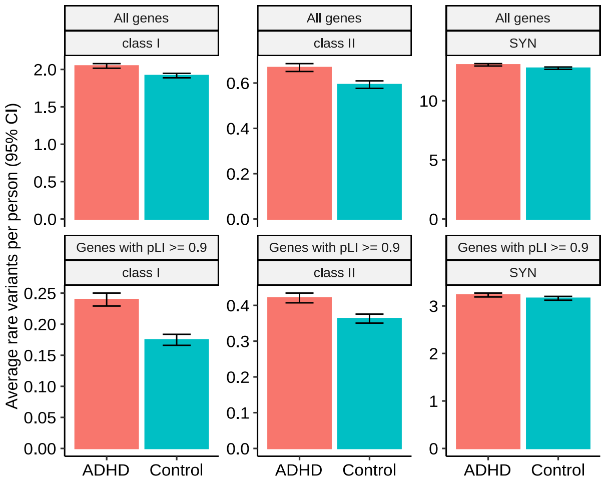

b.

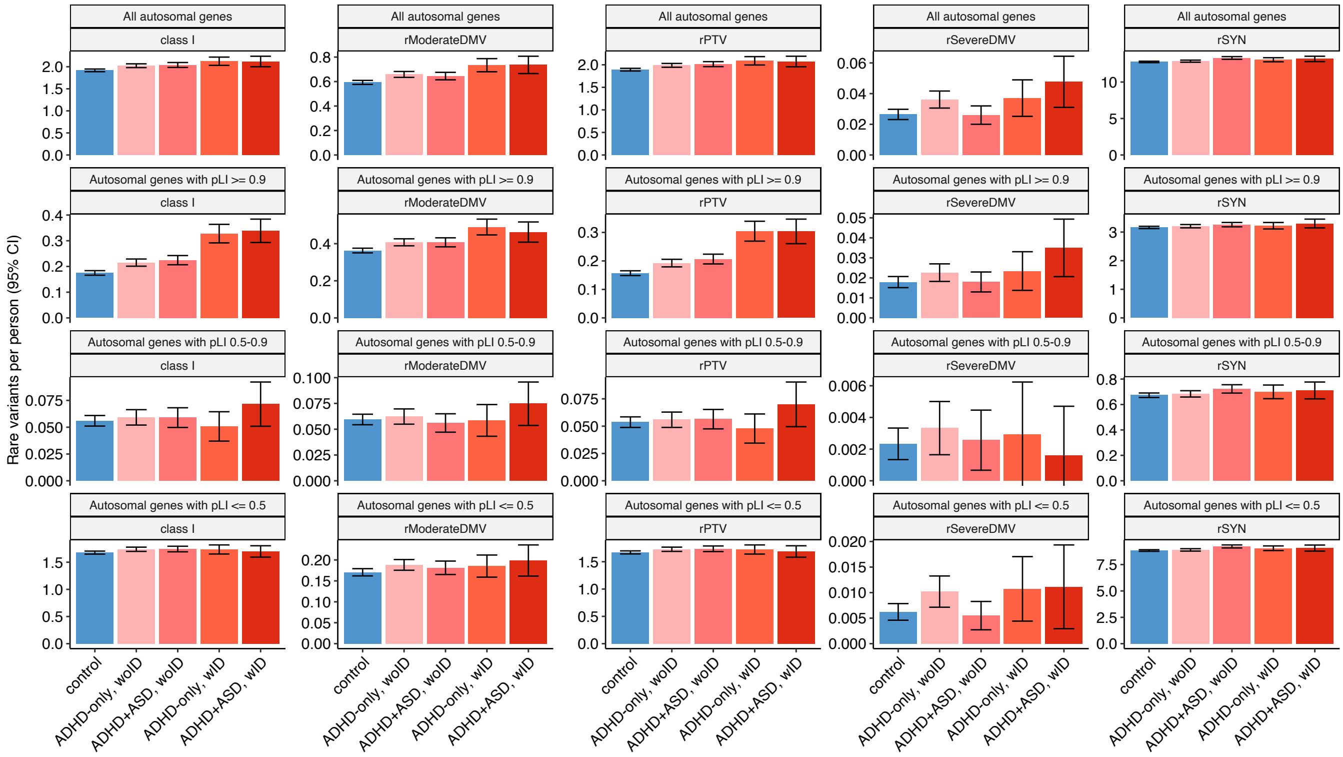

a. Mean count of rare class I, class II and synonymous (SYN) variants in pLI >= 0.9 and in all genes in individuals with ADHD (N = 8,895, red) and controls (N = 9,001, blue). **b.** Mean count of rare variants per individual across ADHD comorbidity subgroups and controls. Mean count of rare class I, rare moderate deleterious missense variants (rModerateDMV), rare protein-truncating variants (rPTV), rare severe deleterious missense variants (rSevereDMV), and rSYN in all autosomal protein-coding genes and in autosomal genes stratified by constraint level (pLI ≥ 0.9, 0.5–0.9, ≤ 0.5). Groups include controls (N = 9,001; blue), individuals diagnosed with ADHD but not ASD and intellectual disability (ID) (ADHD-only, woID, N =4,511; light pink), ADHD with ASD but without ID (ADHD+ASD, woID, N = 2,730; dark pink), ADHD only with ID but not with ASD (ADHD-only, wID, N = 1,025; light red), and ADHD with both ASD and ID (ADHD+ASD, wID, N = 629; dark red). Error bars indicate 95% confidence intervals (CI) of mean count. All numerical values underlying this figure are provided in Supplementary Table 3.

Supplementary Figure 4. Sex-specific rare variant analyses

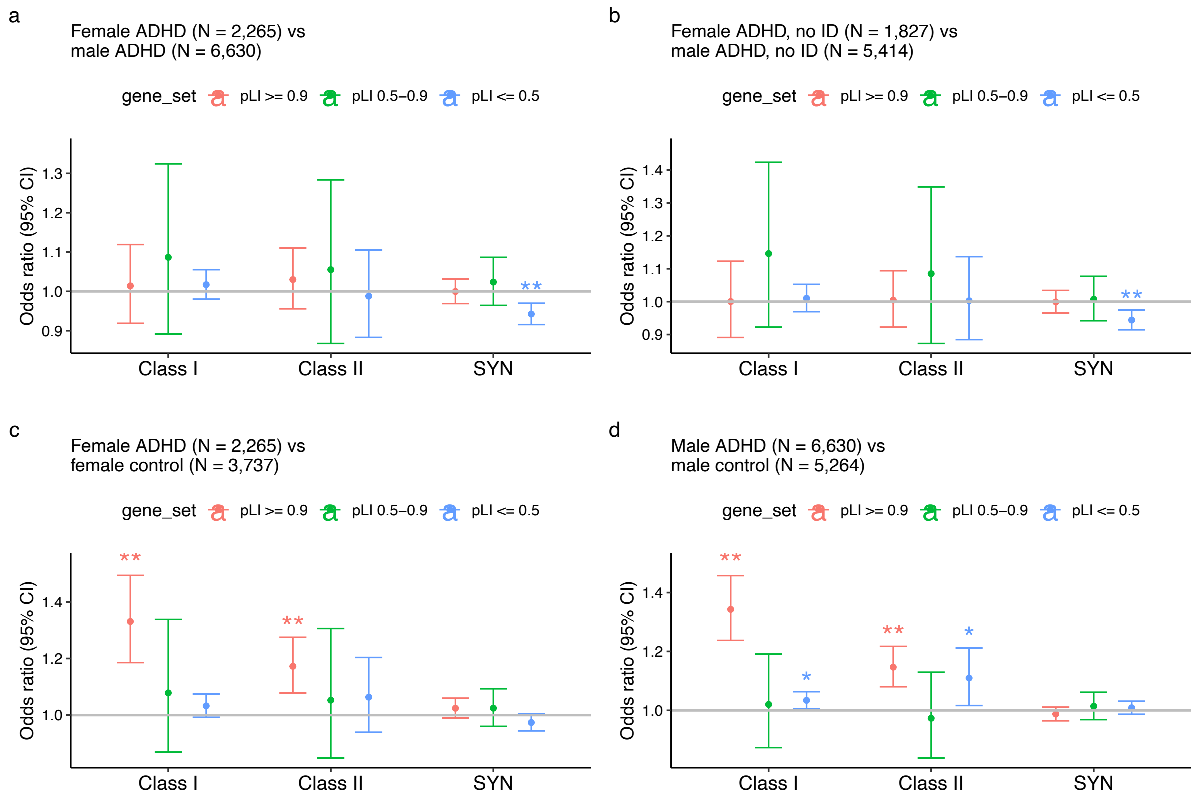

The load of rare class I, class II and rare synonymous variants (SYN) across pLI bins in **a.** females with ADHD (N = 2,265) compared to males with ADHD (N = 6,630) and **b.** females with ADHD (N = 1,827) compared to males with ADHD (N = 5,414) where individuals with ID have been excluded **c.** females with ADHD (N = 2,265) compared to female controls (N = 3,737) **d.** males with ADHD (N = 6,630) compared to male controls (N = 5,264). *Indicates nominal significant association P < 0.05, **Indicates significant association after correction for multiple testing (a P-value = 0.0167 is considered significant, correcting for three gene sets). Each point represents the odds ratio estimate from logistic regression, and error bars represent the 95% confidence interval (CI) of the estimate. P-values are two-sided and derived from logistic regression. Sample sizes are given at the top of each plot.

Supplementary Figure 5. Comorbidities of individuals with class I variants in *MAP1A*, *ANO8* and *ANK2*

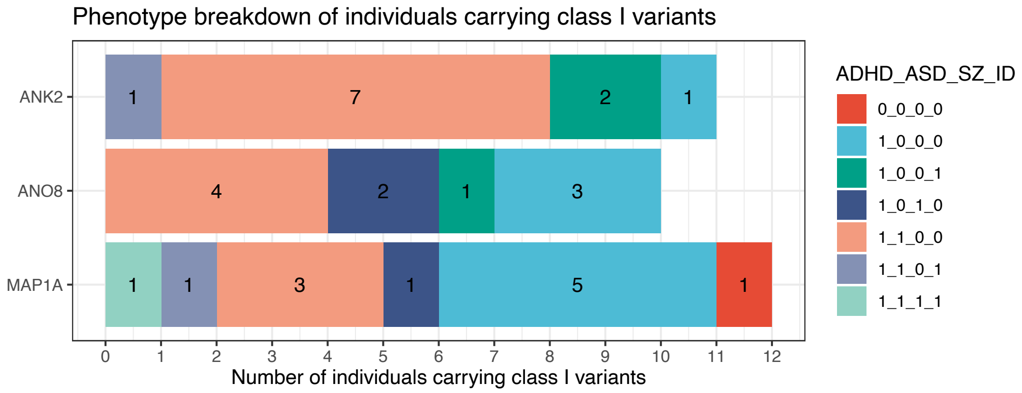

Phenotypic breakdown of individuals carrying at least one rare class I variant. The categories ADHD_ASD_SZ_ID indicate the presence (1) or absence (0) of diagnoses for ADHD, ASD, schizophrenia (SZ), and intellectual disability (ID), respectively. For example, 0_0_0_0 represents controls without any of the listed diagnoses, 1_0_0_0 indicates individuals diagnosed with ADHD only, 1_0_0_1 represents individuals diagnosed with both ADHD and ID, and 1_1_1_1 corresponds to individuals diagnosed with ADHD, ASD, SZ, and ID. Bar colours indicate diagnostic combinations.

Supplementary Figure 6. Ultra-rare variants in clinically ascertained individuals with persistent ADHD across pLI gene sets

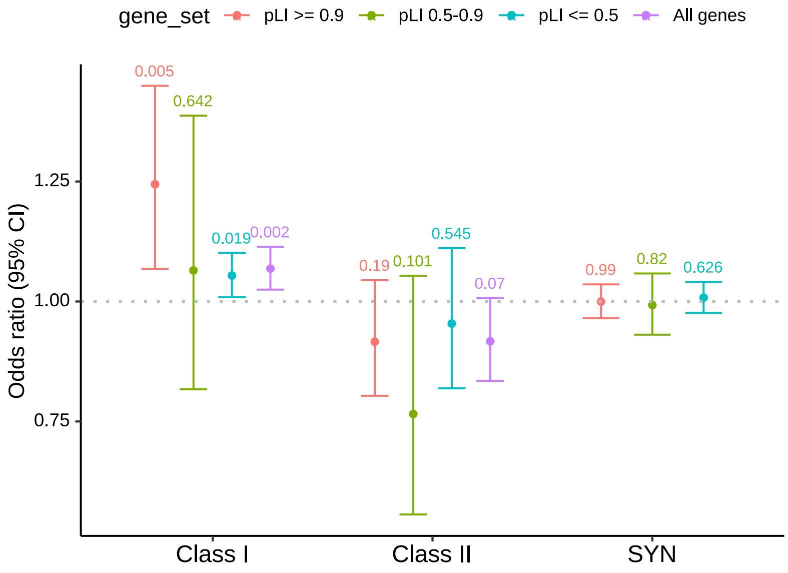

Burden (odds ratios) of ultra-rare class I, class II, and synonymous variants in individuals with clinically ascertained persistent ADHD (N=1,078) compared to controls (N=1,738) across pLI genes sets. Highly constrained genes (pLI ≧ 0.9) marked in red, moderate constrained genes (pLI 0.5 – 0. 9) marked in light green; low constrained genes (pLI ≦ 0.5) marked in blue; all genes marked in purple. Dots represent the odds ratio (OR) estimate from logistic regression, and vertical bars indicate the 95% confidence interval (CI). Two-sided P values from logistic regression are shown above the error bars. All numerical values underlying this figure are provided in Supplementary Table 5.

Supplementary Figure 7. Sex-specific X chromosome rare variant analyses

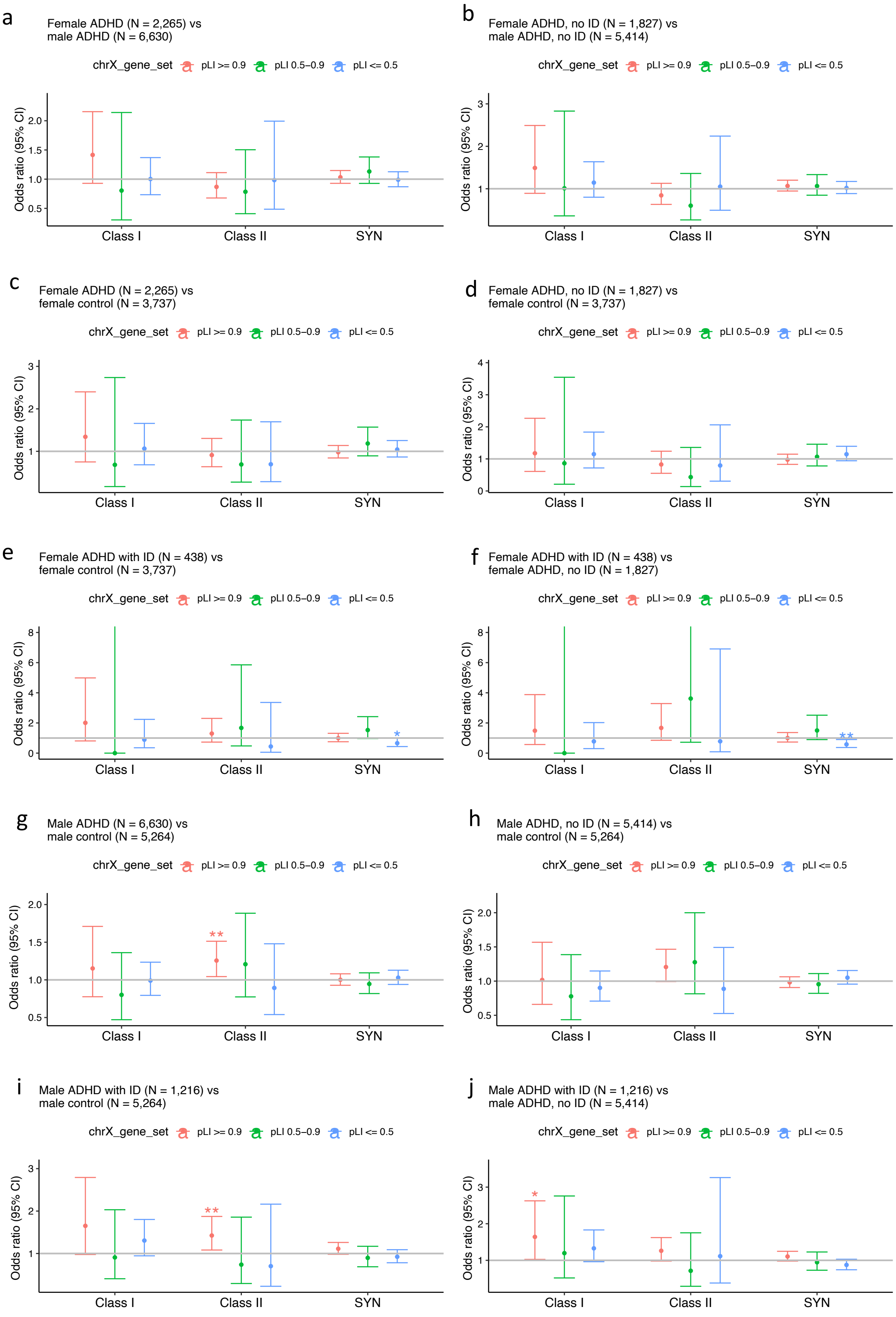

Sex-specific rare variant analyses for chromosome X. The load of rare class I, class II and rare synonymous variants (SYN) across pLI bins of genes on chromosome X in multiple comparisons. Results are from logistic regression. *Indicates nominal significant association P < 0.05, **Indicates significant association after correction for multiple testing (a P-value = 0.0167 is considered significant, correcting for three gene sets). Each point represents the odds ratio estimate from logistic regression, and error bars represent the 95% confidence interval (CI) of the estimate. *P*-values are two-sided and derived from logistic regression. Sample sizes are given at the top of each plot.

Supplementary Figure 8. Expression of ADHD risk genes across brain developmental stages compared to neuronal expressed genes

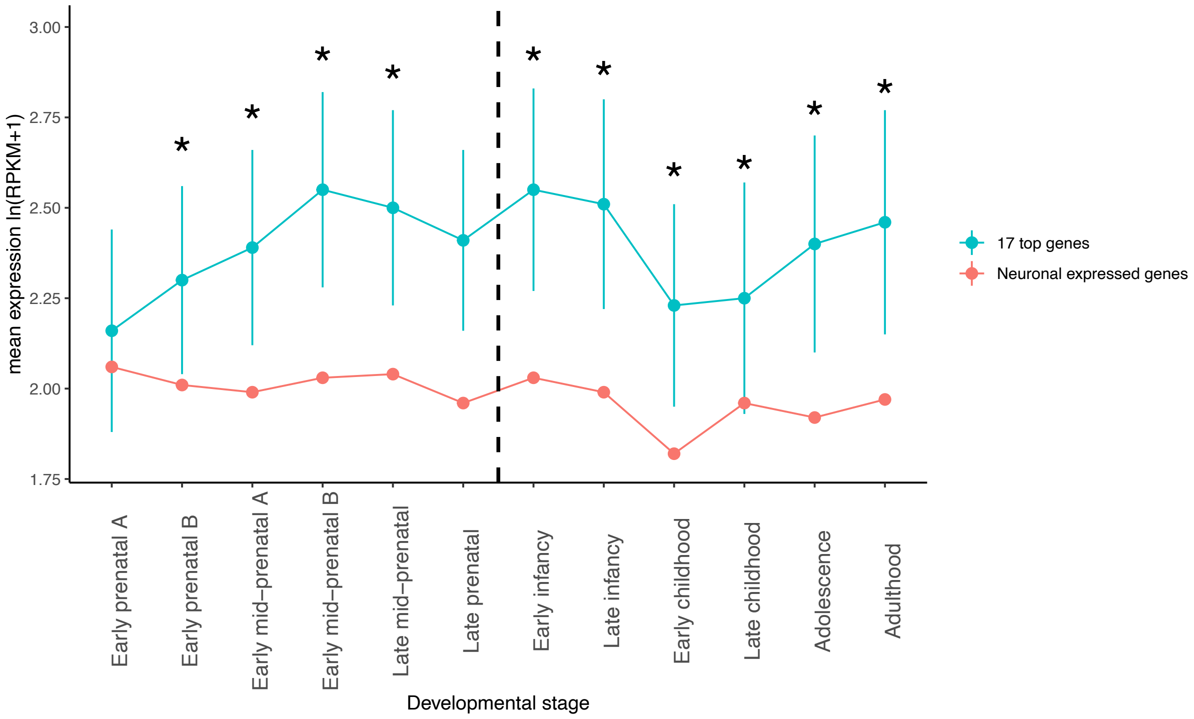

Mean Expression ln(Reads Per Kilobase Million [RPKM] + 1)) of 17 ADHD risk genes (in light blue) and genes expressed in neurons (in red) except the 17 ADHD risk genes, across neocortical brain developmental stages in data from BrainSpan. Genes expressed in neurons are listed in Supplementary PPI-table 7. "*" Indicate significant difference between the two genes sets at a given developmental stage using a two-sided paired t-test (two-sided P-value = 4.17×10^{-3} was considered significant correcting for 12 brain developmental stages) and the vertical bars represent standard errors. All numerical values underlying this figure are provided in Supplementary Table 12.

Supplementary Figure 9. scDRS in cell types from prenatal human midbrain

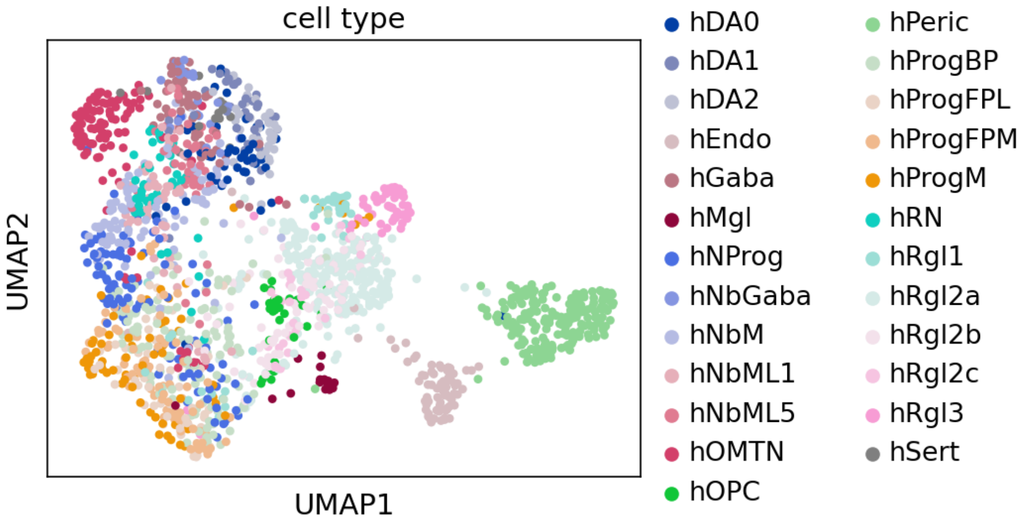

a.

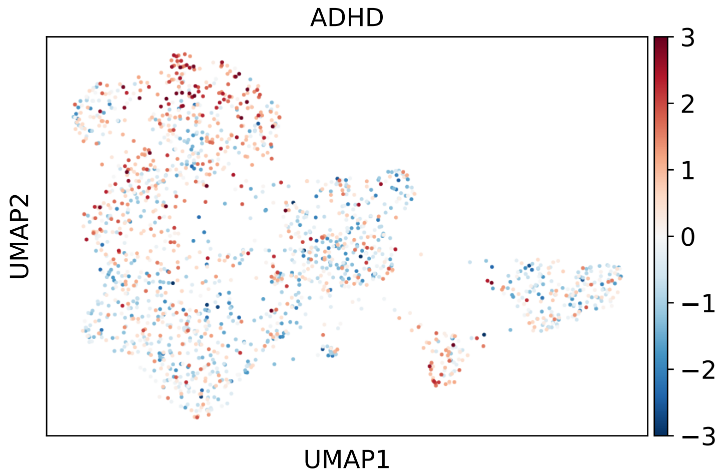

b.

a. UMAP of scRNA-seq data demonstrating clustering of cell types from prenatal human midbrain generated by La Manno et al. (*Cell* **167**, 566-580 e19 (2016)). Dopaminergic neurons type 0h (DA0), Dopaminergic neurons type 1 (hDA1), Dopaminergic neurons type 2 (hDA2), Endothelial cells (hEndo), GABAergic neurons (hGaba), Microglia (hMgl), Neuronal progenitor (hNProg), GABAergic neuroblasts (hNbGaba), Medial neuroblasts (hNbM), Mediolateral neuroblasts 1 (hNbML1), Mediolateral neuroblasts 5 (hNbML5), Oculomotor and trochlear nucleus (hOMTN), Oligodendrocyte precursor cells (hOPC), Pericytes (hPeric), Progenitor basal plate (hProgBP), Progenitor lateral floorplate (hProgFPL), Progenitor medial floorplate (hProgFPM), Progenitor midline (hProgM), Red nucleus (hRN), Radial glia-like cells 1 (hRgl1), Radial glia-like cells 2a(hRgl2a), Radial glia-like cells 2b (hRgl2b), Radial glia-like cells 2c (hRgl2c), Radial glia-like cells 3 (hRgl3), Serotonergic neurons (hSert) **b.** The calculated scDRS for each cell, the strength of the score is indicated by the bar at the right side. Red indicates a positive score reflecting increased expression of ADHD rare variant risk genes compared to the distribution of the expression in control gene sets, blue indicates a negative score reflecting decreased expression of ADHD rare variant risk genes compared to the distribution of the expression of control gene sets.

Supplementary Figure 10. The association of rPTVs with education level and SES in individuals with ADHD and controls

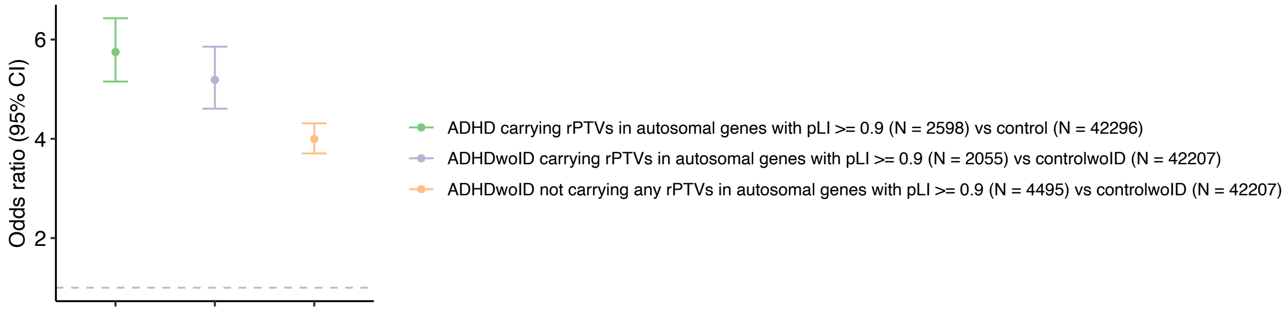

a.

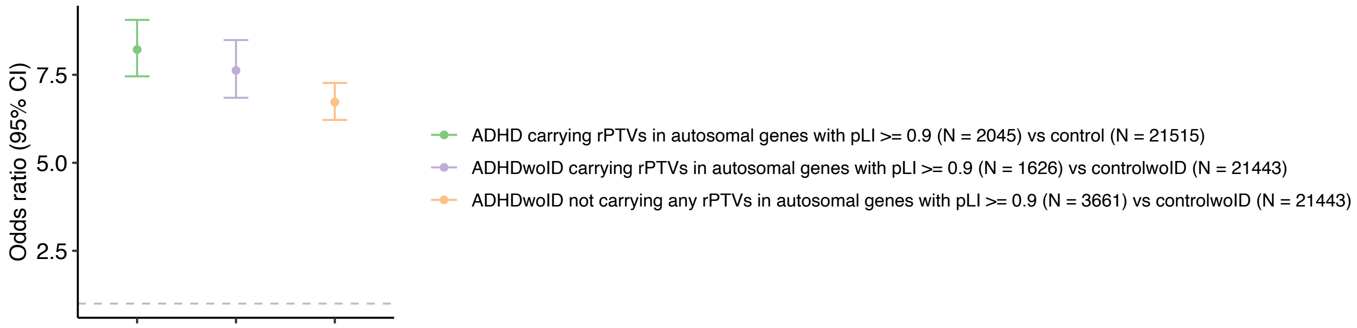

b.

a. Odds ratios for lower education level in individuals with ADHD having ≥ 1 rare protein truncating variant (rPTVs) in constrained autosomal genes (pLI >= 0.9) compared to controls. **b.** Odds ratio of lower SES in individuals with ADHD without intellectual disability (ADHDwoID) having ≥ 1 rPTVs in constrained genes (pLI >= 0.9) compared to controls without intellectual disability (controlswoID). Odds ratio of lower SES in ADHDwoID without rPTVs compared to controls controlswoID. Sample sizes are given next to each figure at the right. Each point represents the odds ratio estimate from logistic regression, and error bars represent the 95% confidence interval (CI) of the estimate. *P*-values are two-sided and derived from logistic regression. The dotted lines represent an odds ratio of 1. All numerical values underlying this figure are provided in Supplementary Table 14.

Supplementary Figure 11. PCA plot of clinically ascertained individuals with persistent ADHD and controls

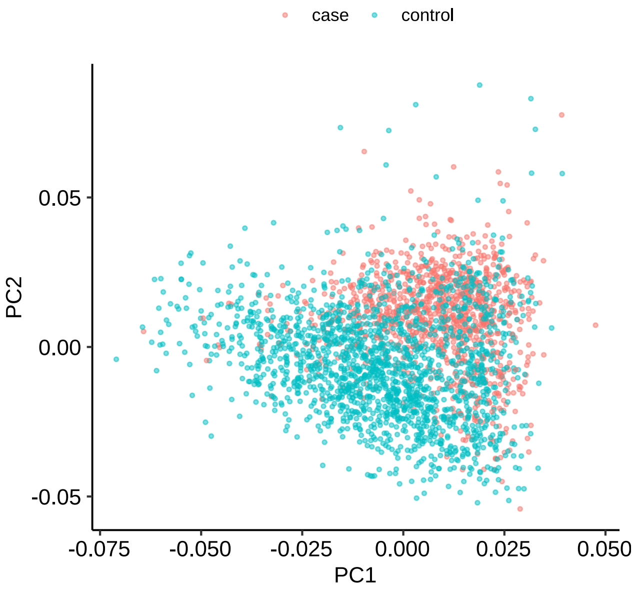

The PCA plot of individuals with clinically ascertained persistent ADHD (N=1,078; marked in red) and controls (N=1,738; marked in light blue) after quality control and removal of genetic outliers. PC, principal component.

Supplementary Figure 12. Quantile-quantile plot of gene-based associations

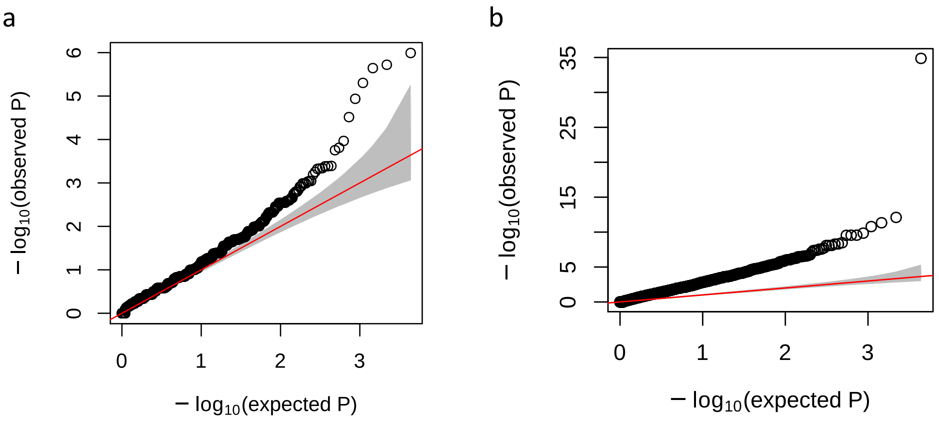

Quantile-quantile (QQ) plots of observed two-sided P-values against expected two-sided P-values from analysis of 8,895 individuals with ADHD and 53,780 controls obtained from (**a**) meta-analysis of combining the impact of both class I and II variants (lambda = 1.53) (**b**) burden test of rare synonymous variants (rSYN). Observed inflation in the rSYN QQ-plot aligns with the anticipatory gene filtering, where genes exhibiting a higher rate of rare synonymous variants in cases compared to controls were selectively excluded during the initial gene discovery phase. Thus, the inflation is caused by less stringent QC in gnomAD demonstrating a consistent increased load of rSYN in controls compared to cases across the genes analysed. The red line indicates the distribution under the null hypothesis and the corresponding standard error.

Supplementary Figure 13. Ultra-rare class I variants in individuals with cardiovascular disease across iPSYCH ADHD risk gene sets

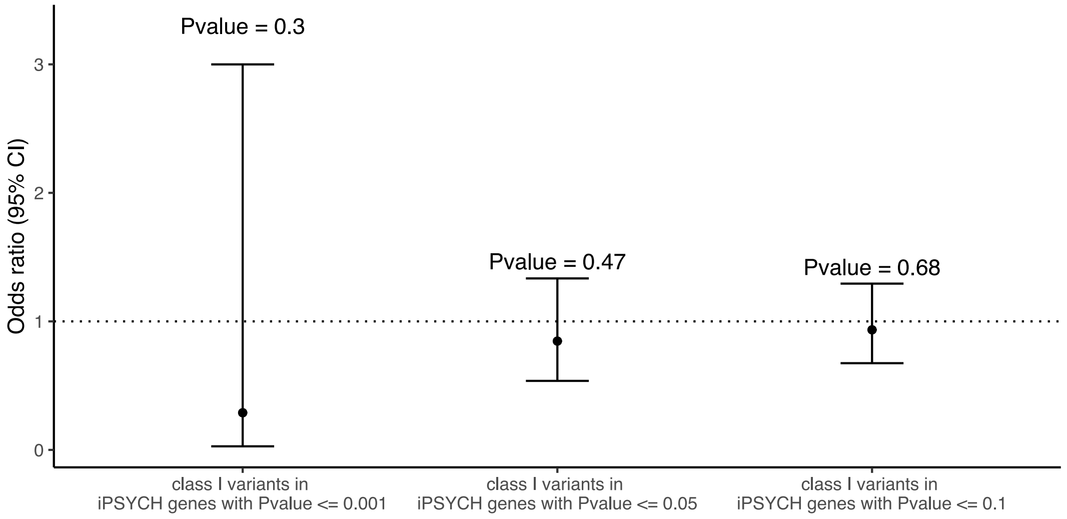

Burden (odds ratio) of ultra-rare class I variants in individuals with cardiovascular disease (N = 825) to those without (N = 838) across gene sets representing genes identified in the gene-based burden test of iPSYCH+gnomAD samples at different P-value thresholds (P ≦ 0.001, P ≦ 0.05, P ≦ 0.1). Each point represents the odds ratio estimate from logistic regression, and error bars represent the 95% confidence interval (CI) of the estimate. *P*-values are two-sided and derived from logistic regression. The dotted lines represent an odds ratio of 1. All numerical values underlying this figure are provided in Supplementary Table 24.

Supplementary Figure 14. iPSC-derived neurons recapitulate features of human neural progenitor cells (NPC) and excitatory neurons (ExN) *in vitro*.

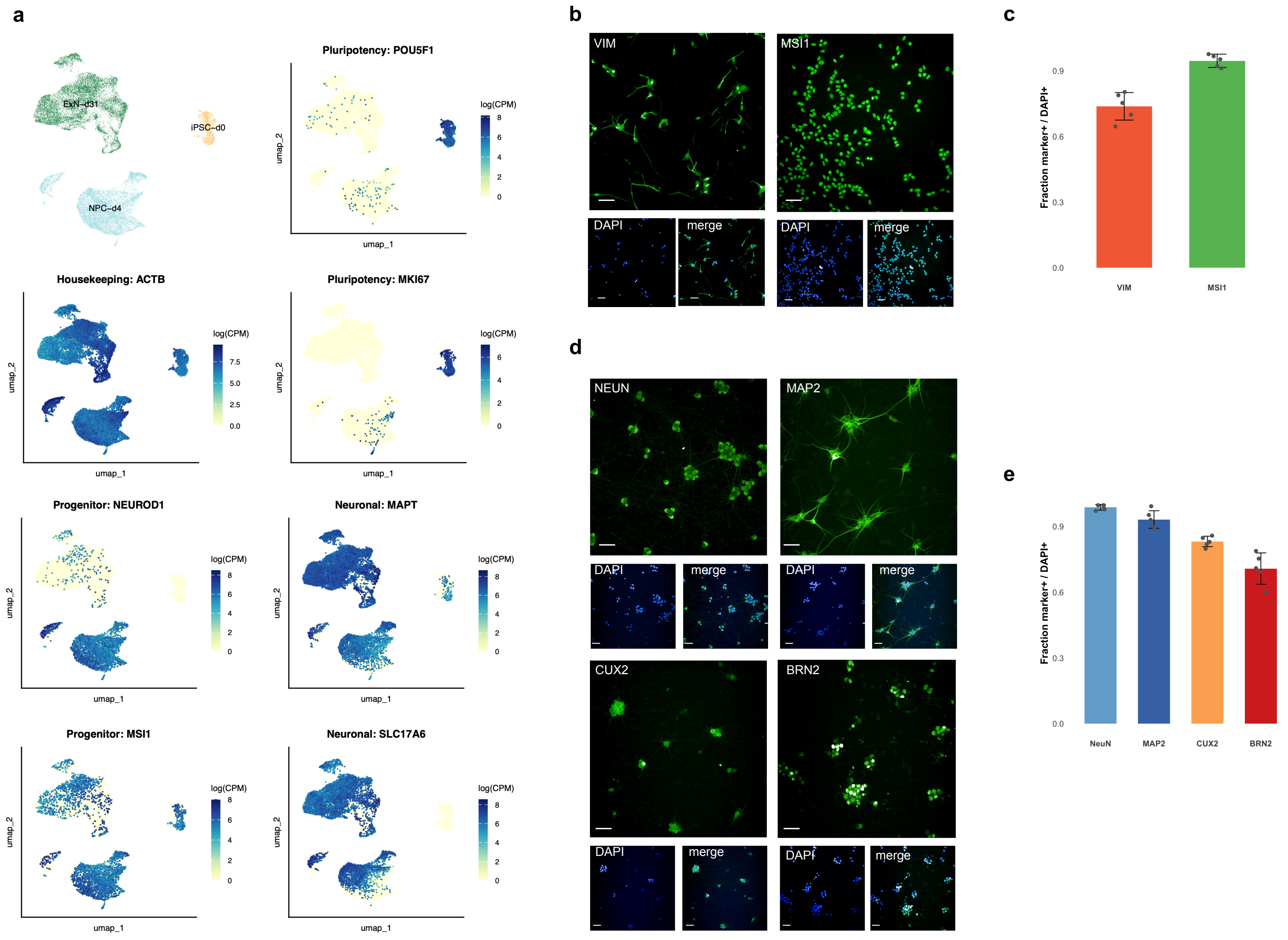

(a) Single-nucleus RNA-seq profiling of iPSC-derived neural cultures reveals expected expression of stage-specific markers: *POU5F1* and *MKI67* for pluripotent cells (iPSC, day 0 of differentiation), *NEUROD1* and *MSI1* for neural progenitors (NPC, day 4), and *MAPT* and *SLC17A6* for excitatory neurons (ExN, day 31). CPM = counts per million reads. **(b)** Immunofluorescence at day 4 of differentiation highlights early neural identity (VIM, MSI1). Scale bar: 50 µm. **(c)** Quantification of five biological replicates (100 cells per replicate) for each IF condition, normalized to cells with DAPI+ nuclei. **(d)** Immunofluorescence at day 30 of differentiation shows expression of mature neuronal markers (NeuN, MAP2) and upper-layer identity (CUX2, BRN2). Scale bar: 50 µm. **(e)** Quantification of five biological replicates (100 cells per replicate) for each IF condition, normalized to cells with DAPI+ nuclei.

References

1 Nehme, R. *et al.* Combining NGN2 Programming with Developmental Patterning Generates Human Excitatory Neurons with NMDAR-Mediated Synaptic Transmission. *Cell reports* **23**, 2509-2523 (2018). https://doi.org:10.1016/j.celrep.2018.04.066

2 Pintacuda, G. *et al.* A foundational neuronal protein network model unifying multimodal genetic, transcriptional, and proteomic perturbations in schizophrenia. *medRxiv* (2025). https://doi.org:10.1101/2025.05.02.25326757

3 Zheng, G. X. *et al.* Massively parallel digital transcriptional profiling of single cells. *Nature communications* **8**, 14049 (2017). https://doi.org:10.1038/ncomms14049

4 Hao, Y. *et al.* Dictionary learning for integrative, multimodal and scalable single-cell analysis. *Nat Biotechnol* **42**, 293-304 (2024). https://doi.org:10.1038/s41587-023-01767-y

5 Andreatta, M. *et al.* Semi-supervised integration of single-cell transcriptomics data. *Nature communications* **15**, 872 (2024). https://doi.org:10.1038/s41467-024-45240-z

6 Rappsilber, J., Ishihama, Y. & Mann, M. Stop and go extraction tips for matrix-assisted laser desorption/ionization, nanoelectrospray, and LC/MS sample pretreatment in proteomics. *Anal Chem* **75**, 663-670 (2003). https://doi.org:10.1021/ac026117i

7 Peng, J. & Gygi, S. P. Proteomics: the move to mixtures. *J Mass Spectrom* **36**, 1083-1091 (2001). https://doi.org:10.1002/jms.229

8 Eng, J. K., McCormack, A. L. & Yates, J. R. An approach to correlate tandem mass spectral data of peptides with amino acid sequences in a protein database. *J Am Soc Mass Spectrom* **5**, 976-989 (1994). https://doi.org:10.1016/1044-0305(94)80016-2

9 Tyanova, S. *et al.* The Perseus computational platform for comprehensive analysis of (prote)omics data. *Nature methods* **13**, 731-740 (2016). https://doi.org:10.1038/nmeth.3901

10 Ritchie, M. E. *et al.* limma powers differential expression analyses for RNA-sequencing and microarray studies. *Nucleic acids research* **43**, e47 (2015). https://doi.org:10.1093/nar/gkv007

11 Pintacuda, G. *et al.* Genoppi is an open-source software for robust and standardized integration of proteomic and genetic data. *Nature communications* **12**, 2580 (2021). https://doi.org:10.1038/s41467-021-22648-5

12 Li, T. *et al.* A scored human protein-protein interaction network to catalyze genomic interpretation. *Nature methods* **14**, 61-64 (2017). https://doi.org:10.1038/nmeth.4083

13 Won, H. *et al.* Chromosome conformation elucidates regulatory relationships in developing human brain. *Nature* **538**, 523-527 (2016). https://doi.org:10.1038/nature19847

14 Sey, N. Y. A. *et al.* A computational tool (H-MAGMA) for improved prediction of brain-disorder risk genes by incorporating brain chromatin interaction profiles. *Nature neuroscience* **23**, 583-593 (2020). https://doi.org:10.1038/s41593-020-0603-0

15 Martin, F. J. *et al.* Ensembl 2023. *Nucleic acids research* **51**, D933-D941 (2023). https://doi.org:10.1093/nar/gkac958

16 Demontis, D. *et al.* Genome-wide analyses of ADHD identify 27 risk loci, refine the genetic architecture and implicate several cognitive domains. *Nature genetics* (2023). https://doi.org:10.1038/s41588-022-01285-8

17 Grove, J. *et al.* Identification of common genetic risk variants for autism spectrum disorder. *Nature genetics* **51**, 431-444 (2019). https://doi.org:10.1038/s41588-019-0344-8

18 Mullins, N. *et al.* Genome-wide association study of more than 40,000 bipolar disorder cases provides new insights into the underlying biology. *Nature genetics* **53**, 817-829 (2021). https://doi.org:10.1038/s41588-021-00857-4

19 Howard, D. M. *et al.* Genome-wide meta-analysis of depression identifies 102 independent variants and highlights the importance of the prefrontal brain regions. *Nature neuroscience* **22**, 343-352 (2019). https://doi.org:10.1038/s41593-018-0326-7

20 Trubetskoy, V. *et al.* Mapping genomic loci implicates genes and synaptic biology in schizophrenia. *Nature* **604**, 502-508 (2022). https://doi.org:10.1038/s41586-022-04434-5
21 Yengo, L. *et al.* Meta-analysis of genome-wide association studies for height and body mass index in approximately 700000 individuals of European ancestry. *Hum Mol Genet* **27**, 3641-3649 (2018). https://doi.org:10.1093/hmg/ddy271
22 de Leeuw, C. A., Mooij, J. M., Heskes, T. & Posthuma, D. MAGMA: generalized gene-set analysis of GWAS data. *PLoS Comput Biol* **11**, e1004219 (2015). https://doi.org:10.1371/journal.pcbi.1004219
23 Genomes Project, C. *et al.* A global reference for human genetic variation. *Nature* **526**, 68-74 (2015). https://doi.org:10.1038/nature15393
