# Supplementary material for: Rare genetic variants confer a high risk of ADHD and implicate neuronal biology
Source: Nature. 2025 Nov 12;649(8098):909–17. doi: 10.1038/s41586-025-09702-8 (PMC12823435; doi:10.1038/s41586-025-09702-8)
Supplement: Supplementary file 2 — Reporting Summary [file 41586_2025_9702_MOESM2_ESM.pdf]

nature portfolio

Corresponding author(s): Ditte Demontis
Anders D. Børglum

Last updated by author(s): Aug 12, 2025

Reporting Summary

Nature Portfolio wishes to improve the reproducibility of the work that we publish. This form provides structure for consistency and transparency in reporting. For further information on Nature Portfolio policies, see our Editorial Policies and the Editorial Policy Checklist.

Statistics

For all statistical analyses, confirm that the following items are present in the figure legend, table legend, main text, or Methods section.

n/a	Confirmed	
☐	☒	The exact sample size (n) for each experimental group/condition, given as a discrete number and unit of measurement
☐	☒	A statement on whether measurements were taken from distinct samples or whether the same sample was measured repeatedly
☐	☒	The statistical test(s) used AND whether they are one- or two-sided<br>*Only common tests should be described solely by name; describe more complex techniques in the Methods section.*
☐	☒	A description of all covariates tested
☐	☒	A description of any assumptions or corrections, such as tests of normality and adjustment for multiple comparisons
☐	☒	A full description of the statistical parameters including central tendency (e.g. means) or other basic estimates (e.g. regression coefficient) AND variation (e.g. standard deviation) or associated estimates of uncertainty (e.g. confidence intervals)
☐	☒	For null hypothesis testing, the test statistic (e.g. F, t, r) with confidence intervals, effect sizes, degrees of freedom and P value noted<br>*Give P values as exact values whenever suitable.*
☒	☐	For Bayesian analysis, information on the choice of priors and Markov chain Monte Carlo settings
☒	☐	For hierarchical and complex designs, identification of the appropriate level for tests and full reporting of outcomes
☒	☐	Estimates of effect sizes (e.g. Cohen's d, Pearson's r), indicating how they were calculated

Our web collection on statistics for biologists contains articles on many of the points above.

Software and code

Policy information about availability of computer code

Data collection	Whole-exome seqeuncing was done using Illumina Nextera capture kit and the Illumina HiSeq sequencer. Alignment of sequence reads to the reference genome and and calling of genotypes were done using BWA v0.7.12 and GATK v.3.4. QC was done in Hail v. 0.1 (https://github.com/hail-is/hail), annotation of variants was done using SnpEff v. 4.3t and SnpSift v. 4.3t.
Data analysis	Quality control of sequencing data was done in Hail 0.1. Logistic regression analyses were done in R 4.1.3. Gene based burden test in the clinical cohort was done using EPACTS v.3.3.0 Genetic risk enrichment for overlap with common variants were done using MAGMA (v1.09). Limma-based47 (v3.54.2) two-tailed two-sample moderated t-test in the Genoppi R package (development branch, v1.1.0) was used to calculate enrichment statistics in IP data. Enrichment in PPI-networks of rare-variant associations was performed with a one-tailed Kolmogorov-Smirnov (KS) test. A two-sided paired t-test was used to test for differential expression in BrainSPAN v10 data. UMAPs were generated using Python v3.10.0 and the single-cell analysis libraries Scanpy v1.9.1 and AnnData v0.8.0. The "arpack" algorithm implemented in Scanpy was used to perform PCA. scDRS v1.0.2 was calcuated using the code that can be found here: https://github.com/martinjzhang/scDRS.). Imputation of common variants was done using EAGLE v2.3.5 and Minimac3, and the Haplotype Reference Consortium panel v1.0 as reference. Polygenic scores were generated using the SBayesR algorithm implemented in LDAK v5.2. The c-alpha test implemented in the 'AssotesteR' v0.1-10 R package was used to test for differences in rare variant distributions.. Burden heritability was estimated using the BHR code v0.5.0-alpha that can be found here: https://github.com/ajaynadig/bhr?tab=readme-ov-file. Transcript quantification was performed using Salmon v1.10.2 with GENCODE v43 reference files. Gene-level counts were summarized using tximport v1.26.1, and lowly expressed or non-protein-coding genes were filtered using the filterByExpr function in edgeR v3.40.2.

For manuscripts utilizing custom algorithms or software that are central to the research but not yet described in published literature, software must be made available to editors and reviewers. We strongly encourage code deposition in a community repository (e.g. GitHub). See the Nature Portfolio guidelines for submitting code & software for further information.

Data

Policy information about availability of data

All manuscripts must include a data availability statement. This statement should provide the following information, where applicable:

- Accession codes, unique identifiers, or web links for publicly available datasets
- A description of any restrictions on data availability
- For clinical datasets or third party data, please ensure that the statement adheres to our policy

iPSYCH data are available from the authors after approval by the iPSYCH Data Access Committee and can only be accessed on the secured Danish server (GenomeDK https://genome.au.dk) as the data are protected by Danish legislation. For data access and correspondence please contact: Ditte Demontis (ditte@biomed.au.dk) or Anders D. Børglum (anders@biomed.au.dk).

The IP-MS data for MAP1A and ANO8 have been deposited to MassIVE with identifier MSV000098548.

URLs
BrainSpan: www.brainspan.org
gnomad v2.1.1: https://gnomad.broadinstitute.org/downloads#v2-lof-curation-results
gnomAD release 2.1.1: https://gnomad.broadinstitute.org/downloads#v2
La Manno et al. scRNA-seq data (GSE76381_EmbryoMoleculeCounts.cef.txt.gz) were obtained from: https://www.ncbi.nlm.nih.gov/geo/query/acc.cgi?acc=GSE76381
Jerber et al. snRNA-seq datasets were obtained (version v310.5281/zenodo.4651413) from: https://zenodo.org/record/4651413#.ZAcbxXbMJEZ
Silletti et al. snRNA-seq data were obtained from the CZ CELLxGENE platform:
https://datasets.cellxgene.cziscience.com/f9ecb4ba-b033-4a93-b794-05e262dc1f59.h5ad
Pintacuda, G. et al. RNA-seq data were obtained from GEO: GSE178896: https://www.ncbi.nlm.nih.gov/geo/query/acc.cgi?acc=GSE178896
HipSci data browser: https://www.hipsci.org/#/lines

Research involving human participants, their data, or biological material

Policy information about studies with human participants or human data. See also policy information about sex, gender (identity/presentation), and sexual orientation and race, ethnicity and racism.

Reporting on sex and gender	Samples were removed if the sex, derieved based on genetic information was inconsistent with the reported sex in the registries. The study included: females with ADHD (N = 2,265); males with ADHD (N = 6,630); female controls (N = 3,737); male controls (N = 5,264). No difference in the number of class I, class II, or synonymous variants was observed between males and females with ADHD, either before or after excluding individuals with ID.
Reporting on race, ethnicity, or other socially relevant groupings	Individuals were grouped according to ICD10 codes obtained from the Danish registries, to test for the impact of rare genetic variants on risk of ADHD and co-morbidities. Individuals with no diagnosis of major psychiatric disorders were grouped as controls.
Population characteristics	Participants were born between 1981 and 2008. Psychiatric diagnoses were conferred by the end of 2016 using data from the Danish Psychiatric Central Research Registry and the Danish Patient Registry. Among individuals diagnosed with ADHD (N = 8,895), the mean age at diagnosis was 12.6 years (standard deviation = 6.26).
Recruitment	34,544 individuals from the iPSYCH cohort were selected for whole-exome sequencing, and from these, we included individuals with an ICD-10 diagnosis of ADHD (F90) in the Danish Psychiatric Central Research Registry and the Danish Patient Registry given before or in 2016; individuals with no diagnosis of major psychiatric disorders (autism, bipolar disorder, schizophrenia, and major depressive disorder) were included as controls. Since the information was drawn from national registries and the blood samples were pulled from the Danish Newborn Screening Biobank there is no bias from self-selection.
Ethics oversight	The iPSYCH study was approved by the Scientific Ethics Committee in the Central Denmark Region (Case No 1-10-72-287-12) and the Danish Data Protection Agency. In accordance with Danish legislation, the Danish Scientific Ethics Committee has, for this study, waived the need for specific informed consent in biomedical research based on existing biobanks. The clinical data were approved by the Ethics Committee at the University of Würzburg (Würzburg, Germany); in The Netherlands, by the regional Ethics Committee (Commissie Mensgebonden Onderzoek: CMO Regio Arnhem—Nijmegen; Protocol number III.04.0403 and 2014/290; ABR: NL47721.091.14), the Institutional Review Board of the Radboud University Medical Center (Nijmegen, The Netherlands). Participants were included at the department of Psychiatry at the Radboud University Nijmegen Medical Centre in Nijmegen, The Netherlands. All participants in the German and Dutch samples provided signed informed consent in accordance with the Declaration of Helsinki.

Note that full information on the approval of the study protocol must also be provided in the manuscript.

Field-specific reporting

Please select the one below that is the best fit for your research. If you are not sure, read the appropriate sections before making your selection.

☒ Life sciences ☐ Behavioural & social sciences ☐ Ecological, evolutionary & environmental sciences

For a reference copy of the document with all sections, see nature.com/documents/nr-reporting-summary-flat.pdf

Life sciences study design

All studies must disclose on these points even when the disclosure is negative.

Sample size	The sample size is based on findings from other studies e.g. in our previous study (Satterstrom et al. Nature Neuroscience, 2019) we analyzed 3,091 individuals with autism spectrum disorder (ASD), 3,206 with ADHD and 684 diagnosed with ASD+ADHD and 5002 ontrols. In the previous study we identifed one significant rare-variant risk gene for ASD+ADHD. In the current study the sample size for individuals with ADHD and controls is higher and thus we expected to have power to identify ADHD risk genes.
Data exclusions	We aimed at analyzing genetically homogeneous individuals. Genetic outliers were excluded based on principal component analyses and related individuals were removed (cases preferred kept over controls).Additionally individuals were excluded if the imputed sex did not match the sex in the registries.
Replication	Generalizability of our findings was assessed in 1,078 individuals with clinical ascertained persistent ADHD and 1,738 controls. We replicated the finding of increased load of class I variants in cases compared to controls. Top genes from our discovery analysis showed a tendency towards higher effect size point estimates than constrained genes.
Randomization	Allocation into groups was not random. Individuals were allocated to a phenotype based on diagnosis codes.
Blinding	In iPSYCH, diagnoses are drawn from registries based on ICD10 diagnosis codes. These are administrative data bases populated by data from the clinicians long before the current study. The blood samples are pulled from a biobank. Hence, the study participants and diagnosing clinicians are blinded with respect to this study. Genotyping is done on a massive scale overall for the full iPSYCH cohort, and the data is generated without a specif goal or effect in mind except for an overall goal of investigating the genetic and environmental effects on psychiatric disorders.

Reporting for specific materials, systems and methods

We require information from authors about some types of materials, experimental systems and methods used in many studies. Here, indicate whether each material, system or method listed is relevant to your study. If you are not sure if a list item applies to your research, read the appropriate section before selecting a response.

Materials & experimental systems

n/a	Involved in the study	
☐	☒	Antibodies
☐	☒	Eukaryotic cell lines
☒	☐	Palaeontology and archaeology
☒	☐	Animals and other organisms
☒	☐	Clinical data
☒	☐	Dual use research of concern
☒	☐	Plants

Methods

n/a	Involved in the study	
☒	☐	ChIP-seq
☒	☐	Flow cytometry
☒	☐	MRI-based neuroimaging

Antibodies

Antibodies used

Each antibody is listed with the following information in order:
Gene ID: Vendor and catalog number; Host and clonality; Usage; Dilution used in immunofluorescence (IF).

NeuN: Abcam, ab104224; Mouse Monoclonal; Primary; 1:1000

MAP2: Abcam, ab183830; Rabbit Polyclonal; Primary; 1:1000

CUX2: Proteintech, 82933-1-RR; Rabbit Polyclonal; Primary; 1:1000

TUJ1: Invitrogen, MA1-118; Mouse Monoclonal; Primary; 1:1000

TUJ1: Invitrogen, 53-4510-82; Mouse Monoclonal; Conjugated Antibody; 1:500

Homer1: Synaptic Systems, 160003; Rabbit Polyclonal; Primary; 1:1000

Synaptophysin: Synaptic Systems, 101308; Guinea Pig; Primary; 1:1000

Vimentin: Abcam, ab24525; Rabbit Polyclonal; Primary; 1:1000

MSI1: Proteintech, 13512-1-AP; Rabbit Polyclonal; Primary; 1:1000

MAP2: Abcam, ab302547; Rabbit Monoclonal; Conjugated Antibody; 1:500

MAP2: Abcam, ab225316; Rabbit Monoclonal; Conjugated Antibody; 1:500

NeuN: NBP1-77686AF488; Rabbit Polyclonal; Conjugated Antibody; 1:500

Goat Anti-Rabbit IgG H&L (Alexa Fluor® 488): Invitrogen, A-11008; Goat Polyclonal; Secondary Antibody; 1:3000

Goat Anti-Mouse IgG H&L (Alexa Fluor® 488): Invitrogen, A28175; Goat Recombinant Superclonal; Secondary Antibody; 1:3000

Goat Anti-Rabbit IgG H&L (Alexa Fluor® 594): Invitrogen, A-11012; Goat Polyclonal; Secondary Antibody; 1:3000

Goat Anti-Mouse IgG H&L (Alexa Fluor® 594): Invitrogen, A-11032; Goat Polyclonal; Secondary Antibody; 1:3000

Goat anti-Chicken IgY (H+L) Secondary Antibody, Alexa Fluor® 647: Invitrogen, A-21449; Goat Polyclonal; Secondary Antibody; 1:3000

Goat anti-Guinea Pig IgG (H+L) Highly Cross-Adsorbed Secondary Antibody, Alexa Fluor® 488: Invitrogen, A-11073; Goat Polyclonal; Secondary Antibody; 1:3000

Goat anti-Guinea Pig IgG (H+L) Highly Cross-Adsorbed Secondary Antibody, Alexa Fluor® 647: Invitrogen, A-21450; Goat Polyclonal; Secondary Antibody; 1:3000

Validation

All antibodies used in this study have been validated by the respective manufacturers as indicated below
NeuN Abcam, ab104224, NeuN Immunocytochemistry/ Immunofluorescence staining of rat brain neurons using mouse Anti-NeuN antibody Rat brain neural cultures stained with ab104224 in pink with ab4674 (chicken polyclonal to GFAP) in green and DNA in blue. ab104224 reveals strong nuclear and distal cytoplasmic staining. It does not stain astrocytes and other non-neuronal cells.

MAP2 Abcam, ab183830, Immunofluorescent analysis of 4% Paraformaldehyde-fixed, 0.1% TritonX-100 permeabilized rat primary neural/glia cells labelling MAP2 with ab183830 at 1/1000 dilution, followed by ab150077 Goat Anti-Rabbit IgG H&L (Alexa Fluor® 488) antibody at 1/1000 dilution (2 µg/mL) (Green). Confocal image showing positive staining in rat primary neuron cell. Confocal scanning Z step was set as 0.3 µm followed by image processing with maximum Z projection. ab11267 Anti-MAP2 mouse monoclonal antibody was used to counterstain tubulin at 1/500 dilution (4 µg/mL) followed by ab150120 Goat Anti-Mouse IgG H&L (Alexa Fluor® 594) at 1/1000 dilution (2 µg/mL) (Red). The Nuclear counterstain was DAPI (Blue).

CUX2 Proteintech, 82933-1-RR, Immunofluorescent analysis of (4% PFA) fixed HeLa cells using CUX2 antibody (82933-1-RR, Clone: 230235G1) at dilution of 1:300 and CoraLite®488-Conjugated AffiniPure Goat Anti-Rabbit IgG(H+L).

TUJ1 Invitrogen, MA1-118, Immunofluorescence analysis of beta III tubulin (green) in the ectoderm derived from human ES cells. Embryoid bodies (EBs) were generated from the H9 embryonic stem cell line (WiCell Research Institute, WA09) using Gibco® KnockOut™ Serum Replacement. After four days in suspension culture, EBs were plated on Geltrex™-coated tissue culture-treated polystyrene and continuously cultured for 21 days. EB cultures were then fixed and permeabilized according to the 3-Germ Layer Immunocytochemistry Kit (Product # A25538) and stained with anti-beta III-tubulin monoclonal antibody (Product # MA1-118, 1:200 dilution, 5 uL/mL final) at 4°C overnight. Secondary staining was completed using Alexa Fluor™ 488-conjugated anti-mouse IgG (Product # A-11001) and DAPI (Product # D1306) for nuclear DNA (blue) for 1 h at room temperature. Images were taken on EVOS® FL Auto Imaging System at 10X magnification.

TUJ1 Invitrogen, 53-4510-82, Knockout of beta-3 Tubulin (TUBB3) was achieved by CRISPR-Cas9 genome editing. Immunofluorescence analysis was performed on wild type U-87 MG cells (panel a,d), U-87 MG Cas9 cells (panels b,e) and U-87 MG beta-3 Tubulin KO cells (panel c,f). Cells were fixed, permeabilized, and labelled with beta-3 Tubulin Monoclonal Antibody, Alexa Fluor® 488 (Product # 53-4510-82) (10 µg/mL). Nuclei (blue) were stained using ProLong™ Diamond Antifade Mountant with DAPI (Product # P36962), and Rhodamine Phalloidin (Product # R415) (1:300) was used for cytoskeletal F-actin (red) staining. Loss of signal (panel c,f) upon CRISPR mediated knockout (KO) confirms that antibody is specific to beta-3 Tubulin (green). The images were captured at 60X magnification.

Homer1 Synaptic Systems, 160003, Immunostaining of a hippocampus neuron with anti-homer (dilution 1 : 500, red) and anti-synaptophysin (cat. no. 101 011, dilution 1 : 500, green). Positive clusters (red) can be found on the postsynaptic neuron juxtaposed to presynaptic nerve terminals (green).

Synaptophysin Synaptic systems, 101308, Indirect immunostaining of PFA fixed rat hippocampus neurons with Guinea pig anti-Synaptophysin 1 (cat. no. 101 308, dilution 1 : 1000, red) and rabbit anti-MAP 2 (cat. no. 188 002, dilution 1 : 1000, green). Nuclei have been visualized by DAPI staining (blue).

Vimentin Abcam, ab24525, Immunocytochemistry/ Immunofluorescence analysis of neuron/glial cultures labeling Vimentin with ab24525 (green) and GFAP with ab7260 (red). Vimentin is the sole cytoplasmic intermediate filament subunit expressed in fibroblasts, microglial and endothelial cells. The flattened cells in the middle of the image which appear green are fibroblasts. Astrocytes may express primarily GFAP, or both GFAP and vimentin, and so appear red (GFAP only) or golden yellow (GFAP and Vimentin). In cells which express both GFAP and vimentin, the two proteins assemble to produce heteropolymer filaments.

MSl1 Proteintech, 13512-1-AP, Immunofluorescent analysis of (4% PFA) fixed HeLa cells using 13512-1-AP (SNRPB2 antibody) at dilution of 1:50 and Alexa Fluor 488-conjugated AffiniPure Goat Anti-Rabbit IgG

MAP2 Abcam, ab302547, Immunohistochemical analysis of 4% PFA-fixed, 0.2% Triton X-100 permeabilized frozen Mouse cerebellum (fresh) tissue labeling MAP2 with ab302547 at 1/100 (5.0 ug/ml) dilution (Green). Positive staining on mouse cerebellum is observed. The nuclear counterstain was DAPI (Blue).

MAP2 Abcam, ab225316, IHC image of MAP2 staining in a section of frozen normal human cerebral cortex. The section was fixed using 10% formaldehyde in 1XPBS for 10 minutes. No antigen retrieval step was performed prior to staining. Non-specific protein-protein interactions were then blocked in TBS containing 0.025% (v/v) Triton X-100, 0.3M (w/v) glycine and 1% (w/v) BSA for 1h at

room temperature. The section was then incubated overnight at +4°C in TBS containing 0.025% (v/v) Triton X-100 and 1% (w/v) BSA with ab225316 at 1/100 dilution (shown in green) and counterstained using ab195884, Rat monoclonal to Tubulin (Alexa Fluor® 647), at 1/250 dilution (shown in red). Nuclear DNA was labelled with DAPI (shown in blue).

Neun NBP1-77686AF488, RBFOX3/NeuN was detected in immersion fixed U-2 OS human osteosarcoma cell line using Rabbit anti-RBFOX3/NeuN Affinity Purified Polyclonal Antibody conjugated to Alexa Fluor® 488 (Catalog # NBP1-77686AF488) (green) at 10 µg/mL overnight at 4C. Cells were counterstained with DAPI (blue). Cells were imaged using a 100X objective and digitally deconvolved

Goat Anti-Rabbit IgG H&L (Alexa Fluor® 88) Invitrogen, Immunofluorescence analysis of Goat anti-Rabbit IgG (H+L) Cross-Adsorbed Secondary Antibody Alexa Fluor® 488 conjugate was performed using HeLa cells stained with alpha Tubulin Rabbit Polyclonal Antibody (Product # PA5-16891). The cells were fixed with 4% paraformaldehyde for 10 minutes, permeabilized with 0.1% Triton™ X-100 for 10 minutes, blocked with 1% BSA for 1 hour and labeled with 2 µg/mL Rabbit primary antibody for 3 hours at room temperature. Goat anti-Rabbit IgG (H+L) Cross-Adsorbed Secondary Antibody Alexa Fluor® 488 conjugate (Product # A-11008) was used at a concentration of 4 µg/mL in phosphate buffered saline containing 0.2% BSA for 45 minutes at room temperature, for detection of alpha Tubulin in the cytoplasm (Panel a: green). Nuclei (Panel b: blue) were stained with DAPI in SlowFade® Gold Antifade Mountant (Product # S36938). F-actin was stained with Rhodamine Phalloidin (Product # R415, 1:300) (Panel c: red). Panel d represents the composite image. No nonspecific staining was observed with the secondary antibody alone (panel f), or with an isotype control (panel e). The images were captured at 60X magnification.

Goat Anti-Mouse IgG H&L (Alexa Fluor® 488), Invitrogen, Immunofluorescence analysis of Goat anti-Mouse IgG (H+L) Secondary Antibody Alexa Fluor® 488 conjugate was performed using HeLa cells stained with alpha Tubulin (236-10501) Mouse Monoclonal Antibody (Product # A11126). The cells were fixed with 4% paraformaldehyde for 10 minutes, permeabilized with 0.1% Triton™ X-100 for 10 minutes, blocked with 1% BSA for 1 hour and labeled with 2 µg/mL Mouse primary antibody for 3 hours at room temperature. Goat anti-Mouse IgG (H+L)/IgM (L) Secondary Antibody Alexa Fluor® 488 conjugate (Product # A28175) was used at a concentration of 1 µg/mL in phosphate buffered saline containing 0.2% BSA for 45 minutes at room temperature, for detection of alpha Tubulin in the cytoplasm (Panel a: green). Nuclei (Panel b: blue) were stained with DAPI in SlowFade® Gold Antifade Mountant (Product # S36938). F-actin was stained with Rhodamine Phalloidin (Product # R415, 1:300) (Panel c: red). Panel d represents the composite image. No nonspecific staining was observed with the secondary antibody alone (panel f), or with an isotype control (panel e). The images were captured at 60X magnification.

Goat Anti-Rabbit IgG H&L (Alexa Fluor® 594), Invitrogen, Immunofluorescence analysis of Goat anti-Rabbit IgG (H+L) Cross-Adsorbed Secondary Antibody, Alexa Fluor 594 (Product # A-11012) was performed using HeLa cells stained with alpha Tubulin Rabbit Polyclonal Antibody (Product # PA5-16891). The cells were fixed with 4% paraformaldehyde for 10 minutes, permeabilized with 0.1% Triton™ X-100 for 10 minutes, blocked with 1% BSA for 1 hour and labeled with 2 µg/mL of rabbit primary antibody for 3 hours at room temperature. Goat anti-Rabbit IgG (H+L) Cross-Adsorbed Secondary Antibody, Alexa Fluor 594 (A-11012) was used at a concentration of 2 µg/mL in phosphate buffered saline containing 0.2 % BSA for 45 minutes at room temperature, for detection of alpha Tubulin in the cytoplasm (Panel a: red). Nuclei (Panel b: blue) were stained with DAPI in SlowFade® Gold Antifade Mountant (Product # S36938). F-actin was stained with Alexa Fluor® 488 Phalloidin (Product # A12379, 1:300) (Panel c: green). Panel d represents the composite image. No nonspecific staining was observed with the secondary antibody alone (panel f), or with an isotype control (panel e). The images were captured at 60X magnification.

Goat Anti-Mouse IgG H&L (Alexa Fluor® 594), Invitrogen, Immunofluorescence analysis of Goat anti-Mouse IgG (H+L) Highly Cross-Adsorbed Secondary Antibody, Alexa Fluor® 594 conjugate was performed using HeLa cells stained with alpha Tubulin (236-10501) Mouse Monoclonal Antibody (Product # A11126). The cells were fixed with 4% paraformaldehyde for 10 minutes, permeabilized with 0.1% Triton™ X-100 for 10 minutes, blocked with 1% BSA for 1 hour and labeled with 2 µg/mL primary antibody for 3 hours at room temperature. Goat anti-Mouse IgG (H+L) Highly Cross-Adsorbed Secondary Antibody, Alexa Fluor® 594 (Product # A-11032) was used at a concentration of 2 µg/mL in phosphate buffered saline containing 0.2% BSA for 45 minutes at room temperature, for detection of alpha Tubulin in the cytoplasm (Panel a: red). Nuclei (Panel b: blue) were stained with DAPI in SlowFade® Gold Antifade Mountant (Product # S36938). F-actin was stained with Alexa Fluor® 488 Phalloidin (Product # A12379), 1:300) (Panel c: green). Panel d represents the composite image. No nonspecific staining was observed with the secondary antibody alone (panel f), or with an isotype control (panel e). The images were captured at 60X magnification.

Goat anti-Chicken IgY (H+L) Secondary Antibody, Alexa Fluor® 647, Invitrogen, Verification of vagus nerve Schwann cell-specific Mpz-EPOR-KO in mice. A Characterization of mouse vagus nerve derived Schwann cells (VNSCs). The identity and purity of SCs were confirmed using IF staining of S100, p75NTR, and Mpz under a fluorescent microscope (ZEISS Apotome 2). The purity of the cultured SCs (99%) was analyzed by double positive staining of DAPI with S100/p75NTR/Mpz markers from 3 independent experiments. Each image represents 3 images from 3 independent experiments. Scale bar: 50A I 1/4m, naEUR%0=aEUR%03. B PCR genotyping with DNA isolated from the vagus nerve and VNSCs (passage zero, SC-P0; passage one, SC-P1) revealed the presence of the 220-bp PCR product, resulting from MpzCre mediated recombination in the Schwann cell of MpzCre-EPORflox/flox mice and not in wild-type (control; 390A bp) and flox/flox (428A bp) mice (naEUR%0=aEUR%03) - Image collected and cropped by CiteAb under a CC-BY license from the following publication: A critical role for erythropoietin on vagus nerve Schwann cells in intestinal motility. BMC Biotechnol (2023) Image collected and cropped by CiteAb from the following publication (https://pubmed.ncbi.nlm.nih.gov/37127673), licensed under a CC BY license.

Goat anti-Guinea Pig IgG (H+L) Highly Cross-Adsorbed Secondary Antibody, Alexa Fluor® 488, Invitrogen, KRT82 is expressed exclusively in anagen.a IF of KRT82 (green) in postnatal (p) mouse skin. Mouse HFs are in telogen at postnatal days 22 and 90 (p22, p90), in anagen at p31, and catagen at p43. Cuticular staining of KRT82 is only observed in the p31 (anagen) HF. White dashed line outlines the hair shaft cuticle. Weak staining observed in mouse epidermis may be a result of green autofluorescent properties of skin epidermal cells. IF staining was repeated at 3 other anagen timepoints and 2 additional telogen timepoints with similar results. b Western blot analysis of whole mouse skin at varying stages of the hair cycle shows that keratin protein is only present in the anagen phase (p31) of the mouse hair cycle. Western blot was repeated with similar results 2 additional times. Source data are provided as a Source data file. Image collected and cropped by CiteAb from the following publication (https://pubmed.ncbi.nlm.nih.gov/35145093), licensed under a CC BY license.

Goat anti-Guinea Pig IgG (H+L) Highly Cross-Adsorbed Secondary Antibody, Alexa Fluor® 647, Invitrogen, Identification of PPNs and of the recorded PPNs that were immunostained for MOR. (A1) sacral parasympathetic nucleus (SPN) located in the mediolateral border

of the gray matter that was identified under a lower magnification with fluorescent illumination. (A2) tetramethylrhodamine-dextran (TMR)-labeled PPNs were identified at a higher magnification with fluorescent illumination. (A3) The neuron shown in (A2), but viewed with infrared illumination during whole-cell recording. (B1,C1,D1) The recorded neuron shown in (A2,A3), filled with biocytin and visualized with FITC-conjugate avidin (green) at various magnifications. (B2,C2,D2) The same section shows TMR immunoreactivity (Alexa 594) and is shown at the same magnification as in (B1,C1,D1), respectively. (B3,C3,D4) Merged images show that the biocytin-filled neuron was the TMR-containing neuron. (D3) The neuron also shows MOR-Immunoreactive (IR) (Alexa 647). (D4) Merged image shows that the biocytin-filled neuron was the TMR-containing neuron that exhibited MOR-IR. Scale bars: 7 µm in (B); 20 µm in (C); 100 µm in (D). Image collected and cropped by CiteAb from the following publication (https://pubmed.ncbi.nlm.nih.gov/26074773), licensed under a CC BY license.

MAP1 Fortis A301-444A, Detection of human MAP1A by western blot of immunoprecipitates. Samples: Whole cell lysate (1 mg for IP, 20% of IP loaded) from HeLa cells. Antibodies: Affinity purified rabbit anti-MAP1A antibody A301-444A used for IP at 3 µg/mg lysate. MAP1A was also immunoprecipitated by rabbit anti-MAP1A antibody A301-445A, which recognizes a downstream epitope. For blotting immunoprecipitated MAP1A, A301-445A was used at 1 µg/ml. Detection: Chemiluminescence with an exposure time of 10 seconds.

ANO8 Biorbyt orb394715, mouse brain tissue were subjected to SDS PAGE followed by western blot with orb394715 (ANO8 antibody) at dilution of 1:500.

Rabbit IgG polyclonal Abcam ab37415, validated by WB in human liver, mouse brain and rat kidney tissue lysates and HeLa, HepG2 and MCF7 whole cell lysates, for ChIP, with ChIP-qPCR for H3K36me2 on the BTF3 locus in NTKO and TKO cells. Methylation enrichment was tested 5 kb upstream of the TSS (5′) and at the TSS (right). ab37415 was used as the isotype contol.

Eukaryotic cell lines

Policy information about cell lines and Sex and Gender in Research

Cell line source(s): We used an induced pluripotent stem cell (iPSC) line (iPS hDFn 83/22 iNgn2#9 [iPS3]) from a neurotypical male donor.

Authentication: The cell line was not authenticated.

Mycoplasma contamination: Cell lines were tested negative for mycoplasma contamination as per cell culture protocols of the Broad Institute

Commonly misidentified lines (See ICLAC register): No commonly misidentified cell lines were used

Plants

Seed stocks: n/a

Novel plant genotypes: n/a

Authentication: n/a
